# Computational modelling of the pro‐ and antiarrhythmic effects of atrial high rate‐dependent trafficking of small‐conductance calcium‐activated potassium channels

**DOI:** 10.1113/JP288659

**Published:** 2025-07-20

**Authors:** Stefan Meier, Dobromir Dobrev, Paul G. A. Volders, Jordi Heijman

**Affiliations:** ^1^ Department of Cardiology, Cardiovascular Research Institute Maastricht (CARIM), Faculty of Health, Medicine, and Life Sciences Maastricht University and Maastricht University Medical Center Maastricht The Netherlands; ^2^ Institute of Pharmacology, West German Heart and Vascular Center University of Duisburg‐Essen Essen Germany; ^3^ Department of Molecular Physiology & Biophysics Baylor College of Medicine Houston Texas USA; ^4^ Department of Medicine and Research Center Montreal Heart Institute and Université de Montréal Montréal Quebec Canada; ^5^ Gottfried Schatz Research Center, Division of Medical Physics & Biophysics Medical University of Graz Graz Austria

**Keywords:** atrial fibrillation, cardiac arrhythmia, computer model, dynamics, ion channel trafficking, SK channels

## Abstract

**Abstract:**

Small‐conductance calcium‐activated potassium (SK) channels are promising targets for atrial‐specific antiarrhythmic therapies, with evidence suggesting tachycardia‐dependent SK‐channel upregulation. However, the dynamics of SK‐channel gating and trafficking in human atrial electrophysiology remain unclear because of experimental limitations, including the availability of human cardiomyocytes and long patch clamp experiments. Although computational models help explore these mechanisms, none integrate SK‐channel trafficking. In the present study, we expanded our K_v_11.1 trafficking model to simulate rate‐dependent SK‐channel trafficking in a human atrial cardiomyocyte model. Calibrated against experimental data, our model replicates time‐ and rate‐dependent SK‐channel function, allowing simulations of SK‐channel trafficking and its effects on action potentials. Tachypacing at 5 Hz increased SK‐channel density, enhancing SK current and shortening action potential duration, with or without calcium buffering. Two‐dimensional tissue simulations with physiological calcium handling showed that tachycardia increased re‐entry duration and ectopic activity. SK‐channel inhibition reduced re‐entry duration but promoted ectopic activity, suggesting a reduction in atrial fibrillation burden rather than complete elimination. Our novel computational model highlights SK channels’ role in re‐entry‐promoting effects of short atrial tachycardia episodes, offering insights into early atrial fibrillation progression and potential antiarrhythmic strategies.

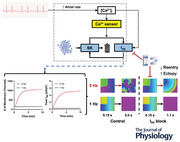

**Key points:**

- Small‐conductance calcium‐activated potassium (SK) channels have emerged as potential targets for atrial‐specific antiarrhythmic therapies, especially in atrial fibrillation (AF).
- Emerging evidence suggests that tachycardia‐induced SK‐channel trafficking can regulate cardiac cellular electrophysiology over minutes, but investigating its impact on arrhythmogenesis in humans is experimentally challenging.
- We adapted our recent *in silico* K_v_11.1 trafficking model to simulate SK‐channel trafficking and incorporated it into a human atrial cardiomyocyte model, which was calibrated based on experimental results.
- Tachypacing at 5 Hz led to a substantial increase in SK channel‐density at the membrane, resulting in enhanced SK current and a reduction in action potential duration.
- 2‐D tissue simulations demonstrated that rapid pacing promoted both re‐entry and ectopic (triggered) activity. Blocking SK channels reduced re‐entry duration but increased ectopic activity, suggesting that SK channel inhibition could decrease AF burden, but may not eliminate AF *per se*.

## Graphical abstract

**Abstract figure legend** Small‐conductance calcium‐activated potassium (SK) channels are important for atrial repolarization and can be targeted for atrial‐specific antiarrhythmic treatments. We developed a computational model with a calcium sensor to study the effects of increased pacing rate (5 Hz), which enhances SK‐channel gating and forward trafficking, amplifying SK current (*I*_SK_), and promoting re‐entry and triggered activity in a two‐dimensional tissue. SK‐channel inhibition reduces re‐entry duration but increases ectopic activity, highlighting their complex role in atrial fibrillation. The Golgi complex, ECG recording and drugs were created with BioRender.com.

## Introduction

Atrial fibrillation (AF) is the most common cardiac arrhythmia, affecting more than 60 million people globally (Linz et al., 2024; Roth et al., 2020). This number is expected to increase significantly in the coming decades as a result of the ageing population (Krijthe et al., 2013). The 2024 ESC Guidelines on AF management introduced the AF‐CARE approach, comprising four key focus areas for optimal AF treatment: managing cardiovascular risk factors and comorbidities, providing anticoagulation therapy to prevent stroke, implementing rate and rhythm control, and continuously evaluating AF progression and treatment effectiveness (Van Gelder et al., 2024). Recent evidence indicates that successful sinus rhythm maintenance using antiarrhythmic drugs (AADs) or catheter ablation can significantly improve clinical outcomes in certain subgroups of AF patients (Boriani et al., 2024; Deering et al., 2024). However, both approaches have suboptimal efficacy and the use of AADs is restricted by the risk of drug‐induced proarrhythmia in patients with advanced structural heart disease (Heijman, Hohnloser et al., 2021). Furthermore, the capacity for AF ablation remains insufficient relative to the growing patient population (Lappalainen et al., 2024). Together, these limitations highlight a substantial need for novel safe and effective AADs. In this context, atrial specificity of drug effects may enhance safety, offering a potential solution to improve treatment outcomes.

One key challenge in managing AF is its progressive nature, famously termed ‘AF begets AF’ (Wijffels et al., 1995). The landmark study by Wijffels et al. (1995) demonstrated that tachycardia‐induced remodelling increases AF inducibility and occurs within hours to days in a goat model. However, recent findings indicate that some components of this remodelling occur on much shorter time scales (i.e. minutes), with specific targets such as small‐conductance calcium‐activated potassium (SK) channels implicated in the process (Heijman et al., 2023; Özgen et al., 2007)

The SK‐channel family (SK1, SK2 and SK3), encoded by the *KCNN1‐3* genes, is predominantly expressed in atrial tissue (Tuteja et al., 2005; Xu et al., 2003), where it contributes to atrial action potential (AP) repolarization through the SK current (*I*_SK_) (Gu et al., 2018; Zhang et al., 2021). SK‐channel activity is regulated by multiple mechanisms. First, SK channels are directly regulated by intracellular calcium concentration ([Ca^2+^]*_i_*) (Adelman et al., 2012), making them highly responsive to rapid pacing or tachyarrhythmias such as AF, which elevate [Ca^2+^]*_i_* (Sun et al., 2001). Second, SK‐channel activity is modulated by post‐translational modification of the Ca^2+^ sensor calmodulin (CaM) (Heijman et al., 2023). Finally, SK channels are also subject to tachycardia‐dependent regulation via trafficking mechanisms, which influence channel density on the cell membrane during rapid pacing (Heijman et al., 2023). Although both reduced and enhanced SK activity have been associated with AF, most studies have shown an increase in *I*_SK_ in patients with AF (Fan et al., 2018; Heijman et al., 2023; Yu et al., 2020), at the same time as reporting a decrease in SK channel mRNA and protein levels (Fan et al., 2018; Rahm et al., 2021; Skibsbye et al., 2014; Yu et al., 2012). This apparent discrepancy between ion current and channel subunit expression levels again highlights the importance of gating and trafficking mechanisms that modulate SK‐channel function independently of expression (Heijman et al., 2023; Saljic et al., 2024).

These results also indicate that *I*_SK_ may play a crucial role in the early phases of AF stabilization, suggesting its potential as a therapeutic target in patients with evolving AF (Saljic et al., 2024). In agreement, a growing body of data highlight the antiarrhythmic effects of SK‐channel inhibition in animal models of persistent AF, where SK‐channel inhibitors prolong atrial repolarization, promote AF termination and reduce AF reinduction. These antiarrhythmic effects have been observed in multiple species, including rats (Skibsbye et al., 2011), dogs (Qi et al., 2014), goats (Gatta et al., 2021), horses (Haugaard et al., 2015) and pigs (Diness et al., 2020, 2017), and persist even in relatively advanced atrial remodelling when currently‐approved AADs such as vernakalant lose their efficacy (Diness et al., 2020, 2017). Notably, the SK‐channel inhibitor AP30663 was recently evaluated in a human phase‐II clinical trial, demonstrating a dose‐dependent cardioversion rate of ∼50% in patients with recent‐onset AF (Holst et al., 2024). Nonetheless, experimental characterization of SK‐channel trafficking and gating is challenging as a result of the limited availability of human tissue samples, technical limitations (such as patch clamp rundown and bleaching during long‐term imaging) and the wide range of timescales involved (from channel gating in milliseconds to trafficking over minutes). Computer models can provide an important solution by offering perfect observability and control over model parameters, allowing researchers to study processes that are otherwise difficult to track experimentally (Heijman, Sutanto et al., 2021; Trayanova et al., 2024). Although recent cardiomyocyte models have provided a novel SK‐channel formulation (Heijman et al., 2023) and an ion‐channel trafficking model for the rapid delayed‐rectifier K^+^ (K_v_11.1) channel (Meier et al., 2023), a model that integrates SK activity, Ca^2+^ handling and dynamic, rate‐dependent SK‐channel trafficking is lacking. In the present study, we developed an SK‐channel trafficking component linked to a Ca^2+^ sensor to simulate the effects of atrial tachycardia‐dependent upregulation of SK‐channel gating and trafficking at the cellular and tissue level to provide insights in the factors contributing to the stabilization of the arrhythmia during the initial minutes of AF.

## Methods

### SK trafficking model

**Figure 1:**
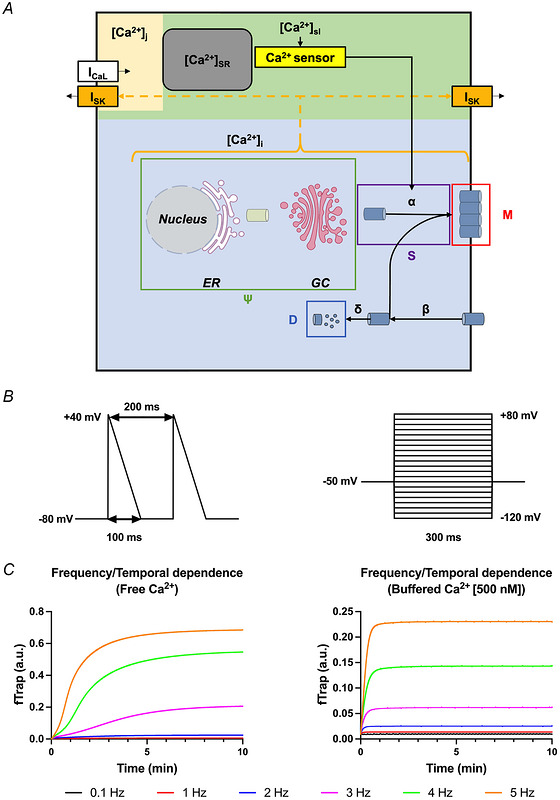
Model components, voltage clamp protocols, and frequency‐dependent temporal dynamics of the Ca^2+^ sensor *A*, the SK‐channel trafficking model, adapted from the K_v_11.1 trafficking model by Meier et al. (2023), consists of two states (M: membrane; S: submembrane) and four rates (ψ: production rate; α: forward trafficking rate; β: internalization rate; δ: degradation rate). Forward trafficking (α) is modulated by Ca^2^⁺ sensor activation, which depends on subsarcolemmal Ca^2^⁺ concentration ([Ca^2^⁺]_sl_). SK‐channel activity is influenced by both [Ca^2^⁺]_sl_ and junctional Ca^2^⁺ concentration ([Ca^2^⁺]_j_). This trafficking framework was integrated into the human atrial cardiomyocyte model (HACM) developed by Grandi et al. (2011) and extended with an SK‐channel formulation developed by Heijman et al. (2023). *B*, two voltage clamp protocols, adapted from Heijman et al. (2023), were implemented using MyoKit (Clerx et al., 2016). The 5 Hz triangular protocol (left) simulates AP‐like stimulation, whereas the square pulse protocol (right) was used for *I*–*V* curve analysis. *C*, the Ca^2^⁺ sensor's frequency and temporal dynamics are illustrated under free (left) and buffered intracellular Ca^2^⁺ conditions (right, fixed at 500 nm). Illustrations of key organelles [nucleus, endoplasmic reticulum (ER), Golgi complex (GC)] were generated using BioRender.com.

We used the human atrial cardiomyocyte model (HACM) as described by Heijman et al. (2023). In short, this model is an extended version of the Grandi et al. (2011) HACM with a novel SK‐channel formulation, *I*_K2P_, along with Na⁺‐dependent regulation of *I*_K1_ and *I*_K,ACh_ (Grandi et al., 2011; Heijman et al., 2023). We extended the HACM with an SK‐channel trafficking component based on our recent model of K_v_11.1 trafficking (Meier et al., 2023), which consists of a submembrane (*S*) state and a membrane (*M*) state with four rates (Fig. 1*A*) modelled by two ordinary differential questions:

(1) $$\frac{dM}{dt}=\left(\alpha \cdot \rho \right)\cdot S-\;\beta \cdot M$$

(2) $$\frac{dS}{dt}=\psi +\beta \cdot M-\left(\alpha \cdot \rho \right)\cdot S-\delta \cdot S$$

where ψ represents the channel production rate, α is the forward trafficking rate, β is the internalization rate and δ is the decay rate (Meier et al., 2023). Given that SK trafficking is both Ca^2+^‐ and rate‐dependent (Heijman et al., 2023), we scaled the forward trafficking rate α by a scalar *ρ’*to introduce the Ca^2+^ dependence of SK‐channel trafficking:

(3) $$\rho =1+\chi \cdot F_{trap}$$

where *F*_trap_ is the relative activation of the Ca^2+^ sensor (described in the next section) and χ is a scalar parameter that controls the maximal absolute upregulation in trafficking rate. The number of membrane channels was determined by dividing the experimentally observed whole‐cell current (*I*_total_) by the single‐channel current (*I*_single_) after 5 Hz stimulation:

(4) $$SK_{mem}=\frac{I_{total}}{I_{single}}$$

With the single‐channel current determined from the product of single‐channel conductance (10 pS) (Kohler et al., 1996) and membrane potential, according to Ohm's law. It is assumed that this current after 5 Hz stimulation represents ∼80% of the total number of SK channels, based on experimental data from Heijman et al. (2023) showing that *I*_SK_ after 5 Hz stimulation in atrial cardiomyocytes from patients without AF becomes comparable to that of patients with AF, which is not significantly affected by 5 Hz stimulation. Based on these data, the total number of SK channels was estimated to be 1165. The final parameters for SK‐channel trafficking can be found in Table 1.

**Table 1:** SK‐channel trafficking parameters

Parameter	Value
α	8.20 × 10^−1^
β	8.29
δ	4.80 × 10^−1^
ψ	5.08 × 10^2^
χ	1.00 × 10^2^

Parameters controlling changes in SK channel numbers are based on a timescale of hours.

### Ca^2+^ sensor controlling SK‐channel trafficking

SK‐channel trafficking is rate‐dependent and closely linked to the [Ca^2+^]*_i_* (Heijman et al., 2023). We used the subsarcolemmal Ca^2+^ concentration ([Ca^2+^]_sl_) as input for a frequency‐dependent Ca^2+^ sensor, with its activity dependent on [Ca^2+^]_sl_ and self‐activation of available sensors by neighbouring sensors that are already activated. In particular, the Ca^2+^ sensitivity was modelled using a Hill function, which increases sensor activity as [Ca^2+^]_sl_ rises:

(5) $$Ca_{dep}=\frac{1}{1+{\left(\frac{K_{m}}{{\left[Ca^{2+}\right]}_{sl}}\right)}^{h}}$$

where *K_m_* represents the Ca^2+^ affinity and *h* is the Hill factor that determines the steepness of the activation curve. The Ca^2+^ sensor's active state was modelled by:

(6) $$active=0.025\cdot \left(1-\;F_{trap}\right)\cdot Ca_{dep}+F_{trap}$$

where *F_trap_* represents the fraction of the Ca^2+^ sensor trapped in the active state, with 1 representing maximal activation. The rate of change of *F*_trap_ over time was modelled as:

(7) $$\frac{dF_{trap}}{dt}=a\cdot active\cdot \left(active-F_{trap}\right)-b\cdot F_{trap}$$

where *a* is the rate at which the sensor becomes trapped in an active state by neighbouring sensors and *b* is the rate of deactivation in the absence of Ca^2+^. The final rate configurations for the Ca^2+^ sensor can be found in Table 2.

**Table 2:** Ca^2+^ sensor parameters

Parameter	Value
*a*	5.00 × 10^−2^
*b*	3.50 × 10^−4^
*K_m_*	1.15 × 10^−3^
*h*	4.00

Parameters controlling changes in Ca^2^⁺ sensor activity are based on a timescale of milliseconds, where applicable.

### Embedding in the human atrial cardiomyocyte model

The above‐mentioned model components with their respective formulas and parameters were embedded in the extended HACM by Heijman et al. (2023). The effects of Ca^2+^‐dependent SK‐channel trafficking on the junctional, subsarcolemmal and total SK current (*I*_SKj_, *I*_SKs_ and *I*_SK_, respectively) were captured as follows:

(8) $$I_{SKj}=F_{j}\cdot g_{SK}\cdot \varepsilon_{SKj}\cdot \eta_{SKv}\cdot \left(V_{m}-E_{K}\right)$$

where *F_j_* is the fractional distribution of SK channels in the junctional region and *g_SK_* is the baseline conductance of SK channels. The *ε_SKj_* is the direct Ca^2+^‐dependent channel activation and *η_SKv_* is the voltage dependence of the SK channels in the junctional space, which together determine the open probability. The difference between the membrane potential (*V_m_*) and the reversal potential of potassium ions (*E_K_*) represents the driving force. For the subsarcolemmal SK current, the current formulation was:

(9) $$I_{SKs}=\left(1-F_{j}\right)\cdot g_{SK}\cdot \varepsilon_{SKs}\cdot \eta_{SKv}\cdot \left(V_{m}-E_{K}\right)$$

where *ε_SKs_* is the direct Ca^2+^‐dependent channel activation of the subsarcolemmal SK channels. The total SK current is determined by the product of both regional SK currents and the relative fraction of channels in the membrane:

(10) $$I_{SK}=\left(\frac{M}{M_{ref}}\right)\cdot \left(I_{SKj}+I_{SKs}\right)$$

where *M* represents the number of SK channels in the membrane and *M*_ref_ represents the number of SK membrane channels in steady state at baseline. The final rate configurations for the SK channel and the additional equations can be found in Table 3.

**Table 3:** SK channel parameters and equations

Parameter	Value
p1	1.01 × 10^−1^
p2	2.73 × 10^−1^
p3	2.96
p4	2.00 × 10^−1^
p5	2.79 × 10^−1^
p6	−8.69 × 10^1^
p7	6.36 × 10^−3^
p8	5.23
p9	3.21 × 10^−4^
g_SK_	3.04 × 10^−2^
F_j_	1.10 × 10^−1^
E_K_	2.67 × 10^1^
M_ref_	1.05 × 10^2^
SK channel equations	
$\;\varepsilon_{SKj}=\frac{1}{1+\exp(\frac{log_{10}\left(p9\right)-log_{10}\left({\left[Ca^{2+}\right]}_{j}\right)}{0.3})}$	
$\;\varepsilon_{SKs}=\frac{1}{1+\exp(\frac{log_{10}\left(p9\right)-log_{10}\left({\left[Ca^{2+}\right]}_{sl}\right)}{0.3})}$	
$\;\eta_{SKv}=\frac{p2}{1+\exp((V_{m}-E_{K}+p3)\cdot p4)}+\frac{p5}{1+\exp((-(V_{m}-E_{K}+p6))\cdot p7)}$	

Parameters controlling SK channel activity are based on a timescale of milliseconds, where applicable.

### Cellular voltage clamp protocols

To replicate the experimental conditions during which [Ca^2+^]*_i_* was buffered with ethylene glycol‐bis(β‐aminoethyl ether)‐*N*,*N*,*N*′,*N*′‐tetra acetic acid (EGTA), the cytosolic Ca^2+^ was fixed at 500 nm in the model, whereas junctional and sarcolemmal Ca^2+^ levels were kept dynamic, to account for the relatively slow buffering characteristics of EGTA (Eisner et al., 2023). After parameter optimization, all simulations were performed with free [Ca^2+^]*_i_* concentrations (unbuffered), unless stated otherwise.

Heijman et al. (2023) used several voltage clamp protocols to experimentally characterize *I*_SK_ and SK‐channel trafficking, which were replicated in the *in silico* model. A triangular voltage clamp protocol was used to mimic rapid electrical activation at various frequencies over longer periods (i.e. minutes). In this protocol, the membrane was held at −80 mV, depolarized to +40 mV, and then gradually returned to −80 mV over 100 ms. This protocol was then repeated for a given frequency and time period (Fig. 1*B*, left). For the square voltage clamp protocol, cells were held at a membrane potential of −50 mV, followed by voltage steps ranging from −120 mV to +80 mV in 10 mV increments, with each step lasting 300 ms, before returning to the holding potential of −50 mV (Fig. 1*B*, right). This protocol was used to generate current–voltage (*I*–*V*) curves.

### Tissue simulation protocol

The two‐dimensional (2‐D) tissue simulations were conducted in a 6 × 6 cm homogenous piece of virtual atrial tissue represented by 600 × 600 cellular units, with an isotropic conduction velocity of ∼57 cm s^−1^. All simulations were initialized using single‐cell steady‐state conditions obtained after 1 Hz current clamp pacing for 1000 beats. Subsequently, the single HACM was subjected to activation by the current clamp protocol at 1 Hz for 10 min, simulating sinus rhythm. This was followed by stimulation at 5 Hz for 10 min to mimic a brief episode of (paroxysmal) AF, after which the pacing frequency was returned to 1 Hz for an additional 10 min, reflecting cardioversion to sinus rhythm. The tissue simulations were performed using the cellular states recorded at several time points: 1 min before, 3 min after and 9 min after the initiation of 5 Hz stimulation, and 3 min after the stimulation was switched back to 1 Hz. In this way, we could study the effects of frequency‐dependent SK‐channel trafficking on arrhythmia inducibility and stability. An S_1_S_2_ stimulation protocol, similar to that used in our previous study (Bodi et al., 2024), was implemented to evaluate the inducibility and stability of re‐entry. The first stimulus (S_1_) induced a planar excitation wave traveling from left to right, while the second stimulus (S_2_) was delivered to the upper‐left quadrant of the tissue at varying coupling intervals. When the tissue was sufficiently recovered from the S_1_ activation, the S_2_ stimulus could initiate a reentrant wave. Arrhythmogenesis was quantified by measuring the re‐entry duration (in milliseconds) and summing the re‐entry duration over all the tested S_1_S_2_ intervals for each condition. Furthermore, the occurrence of ectopic (triggered) activity [i.e. delayed afterdepolarizations (DADs) or triggered APs] was also used as a marker for arrhythmogenesis. The simulations were repeated under conditions of 80% SK channel block to assess its impact on atrial arrhythmogenesis.

### Statistics, software and data availability

The experimental data are presented as the mean ± SD. All simulations were performed through Myokit and Python [versions 3.8.10 (tissue simulations) and 3.9.6 (cellular simulations)] (Clerx et al., 2016). The model code, optimization scripts and data are available online (https://github.com/HeijmanLab) and an archived, citable snapshot of the specific version used in this study is accessible via Zenodo (https://doi.org/10.5281/zenodo.15494834).

## Results

### Frequency and temporal dynamics of the SK‐channel trafficking model

Upregulation of SK‐channel trafficking during 5 Hz activation is [Ca^2+^]_i_‐dependent (Heijman et al., 2023). As such, we implemented a novel Ca^2^⁺ sensor in our model to simulate the dynamic frequency‐dependent changes in SK‐channel membrane levels. The sensor is activated by subsarcolemmal [Ca^2+^] and exhibits a slow deactivation, resulting in accumulation of activity at rapid rates. After 10 min of triangular voltage clamp pulses at different frequencies, both the buffered and free Ca^2+^ conditions showed increased Ca^2^⁺‐sensor activation with faster activation frequencies (Fig. 1*C*). However, the free Ca^2+^ simulations displayed a more dynamic and larger increase in Ca^2+^‐sensor activity (Fig. 1*C*, left) than the buffered condition, which plateaus within the first minute resulting in a diminished frequency response above 2 Hz (Fig. 1*C*, right).

### SK‐channel model calibration under buffered [Ca^2+^]_i_

**Figure 2:**
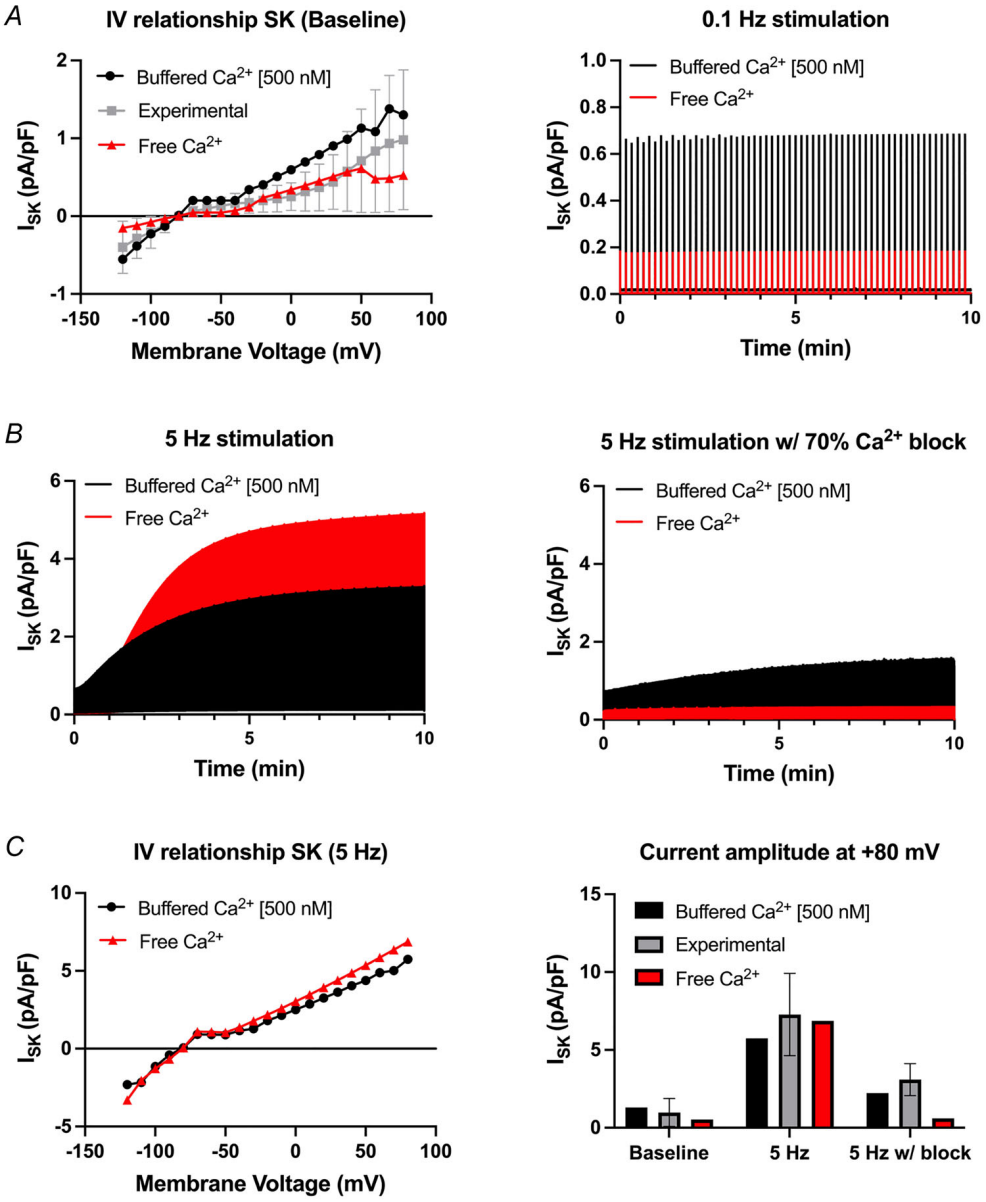
Calibration of the SK‐channel model *A*, the SK current–voltage (*I*–*V*) relationship was calibrated using the square‐pulse voltage clamp protocol from Fig. 1*B* (right), under both buffered intracellular Ca^2^⁺ (fixed at 500 nm) and free Ca^2^⁺ conditions. Simulated results were compared to the experimental *I*–*V* curves (mean ± SD) from Heijman et al. (2023). Right: *I*_SK_ traces following 10 min of stimulation with the triangular voltage clamp protocol at 0.1 Hz. *B*, the increase in *I*_SK_ after 10 min of triangular stimulation at 5 Hz for both free and buffered intracellular Ca^2^⁺ conditions without (left) or with (right) 70% l‐type Ca^2^⁺ channel block (simulating the effects of 1 µmol L^−1^ nifedipine (Heijman et al., 2023; Shen et al., 2000)). *C*, Post 5 Hz stimulation *I*–*V* relationships for both free and buffered Ca^2^⁺ conditions (left) and overview of SK‐current amplitudes at +80 mV for baseline, post 5 Hz stimulation, and post 5 Hz stimulation with LTCC block (right). Experimental data and SDs are included for comparison (Heijman et al., 2023).

The model was optimized based on experimental data from Heijman et al. (2023), which were collected under buffered [Ca^2+^]*_i_*, at baseline (i.e. during a square voltage clamp protocol at the start of the experiment) or after high‐rate (i.e. 5 Hz triangular voltage clamp protocol) activation for 10 min, with or without inhibition of the l‐type Ca^2+^ channel (LTCC). To ensure close alignment with these data, we closely mimicked the experimental protocols in our simulations and parameter optimization processes. Under baseline conditions (i.e. square‐pulse voltage clamp simulation), the simulated *I*–*V* relationship under buffered Ca^2^⁺ (500 nmol L^−1^) aligned well with the experimental *I*–*V* curves (Fig. 2*A*, left). When the triangular AP‐like voltage clamp protocol was applied for 10 min at a low frequency (0.1 Hz), *I*_SK_ remained stable (Fig. 2*A*, right), consistent with the frequency dependence of the Ca^2+^ sensor shown in Fig. 1*C*. By contrast, following 10 min of triangular stimulation at 5 Hz, the model exhibited a ∼5‐fold increase in *I*_SK_ under buffered Ca^2^⁺ conditions (Fig. 2*B*, left). However, simulating a 70% LTCC block (representing the effect of 1 µmol L^−1^ nifedipine used experimentally (Heijman et al., 2023; Shen et al., 2000)) strongly suppressed this frequency‐dependent upregulation of *I*_SK_ (Fig. 2*B*, right). The *I*–*V* curves obtained after 10 min of triangular stimulation at 5 Hz also display a significant increase in *I*_SK_ relative to the baseline *I*–*V* curves under buffered Ca^2^⁺ conditions (Fig. 2*C*, left). Figure 2*C* (right) summarizes these results, confirming that the model can reproduce the buffered experimental data for baseline, 5 Hz stimulation and LTCC block conditions.

### SK‐channel gating and trafficking under free [Ca^2+^]_i_

After calibrating the model under buffered Ca^2^⁺ conditions, we extended the simulations to free [Ca^2+^]*_i_*. The baseline *I*–*V* curves from these simulations remained within experimental SDs but exhibited a less steep *I*–*V* relationship compared to buffered conditions, underscoring the impact of Ca^2^⁺ buffering on I_SK_ (Fig. 2*A*, left). Similar to the buffered conditions, *I*_SK_ remained stable following 10 min of low‐frequency (0.1 Hz) triangular stimulation, albeit with a much smaller maximal *I*_SK_, again emphasizing the role of direct Ca^2+^‐dependent SK‐channel activation as a result of buffering [Ca^2+^]*_i_* at 500 nmol L^−1^ (Fig. 2*A*, right). After 10 min of high‐frequency (5 Hz) triangular stimulation, *I*_SK_ under free Ca^2^⁺ was more than 50% higher than under buffered conditions and showed an ∼30‐fold increase compared to low‐frequency stimulation (Fig. 2*B*, left). The effects of LTCC block were also more pronounced under free Ca^2^⁺, with a stronger reduction in *I*_SK_ (Fig. 2*B*, right). By contrast, the *I*–*V* curves after 5 Hz stimulation did not display the striking differences in peak *I*_SK_ observed during the triangular stimulation (Fig. 2*C*, left) because of a reduction in Ca^2+^ loading during the longer intervals of the square‐pulse *I*–*V* protocol compared to the triangular protocol, affecting SK‐channel gating. Overall, free Ca^2^⁺ conditions resulted in greater frequency‐dependent *I*_SK_ upregulation following high‐frequency stimulation compared to buffered Ca^2^⁺, although values remained within the experimental SD (Fig. 2*C*, right). Subsequent LTCC block caused a sharp reduction in *I*_SK_ (Fig. 2*C*, right). These results emphasize the enhanced frequency‐dependent upregulation of *I*_SK_ under free Ca^2^⁺ as a result of both Ca^2+^‐dependent gating and trafficking mechanisms, which suggest a potential role for *I*_SK_ upregulation in modulating arrhythmia stability during episodes of paroxysmal AF.

### Sensitivity analysis of Ca^2+^‐sensor parameters

**Figure 3:**
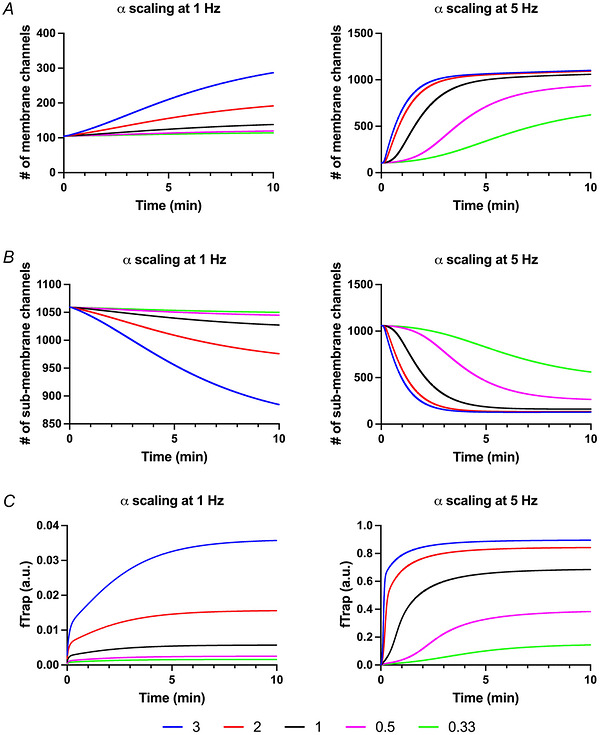
Sensitivity of SK channel behaviour to changes in the Ca^2^⁺ sensor activation rate (α) *A*, effect of scaling (3‐, 2‐, 1‐, 0.5‐ or 0.33‐fold) α on the number of SK channels in the membrane and their dynamics after 10 min of triangular voltage clamp stimulation at either 1 Hz (left) or 5 Hz (right). *B*, corresponding changes in the number of submembrane SK channels under identical conditions. *C*, activation and dynamic behaviour of the Ca^2^⁺ sensor at different α values, highlighting the rate‐dependent differences between 1 Hz (left) and 5 Hz (right) stimulations.

**Figure 4:**
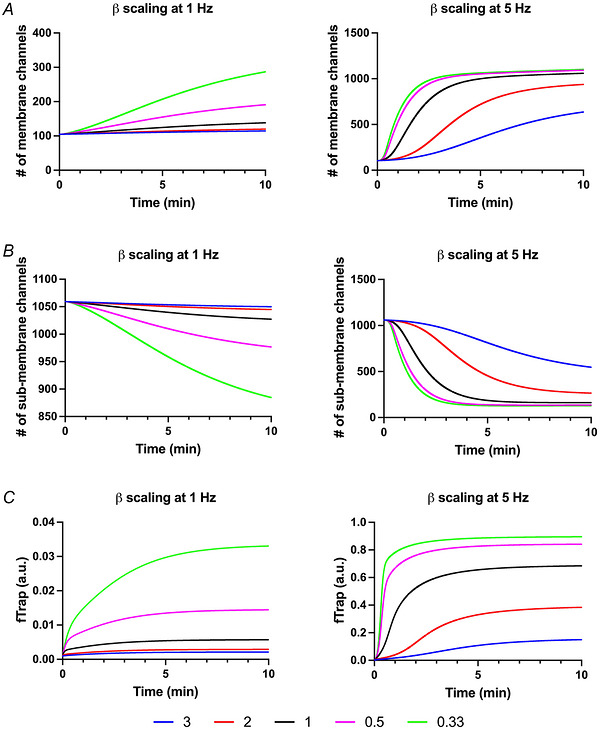
Sensitivity of SK channel behaviour to changes in the Ca^2^⁺ sensor de‐activation rate (β) *A*, effect of scaling (3‐, 2‐, 1‐, 0.5‐ or 0.33‐fold) β on the number of SK channels in the membrane and their dynamics after 10 min of triangular voltage clamp stimulation at either 1 Hz (left) or 5 Hz (right). *B*, Corresponding changes in the number of submembrane SK channels under identical conditions. *C*, activation and dynamic behaviour of the Ca^2^⁺ sensor at different β values, highlighting the rate‐dependent differences between 1 Hz (left) and 5 Hz (right) stimulations.

**Figure 5:**
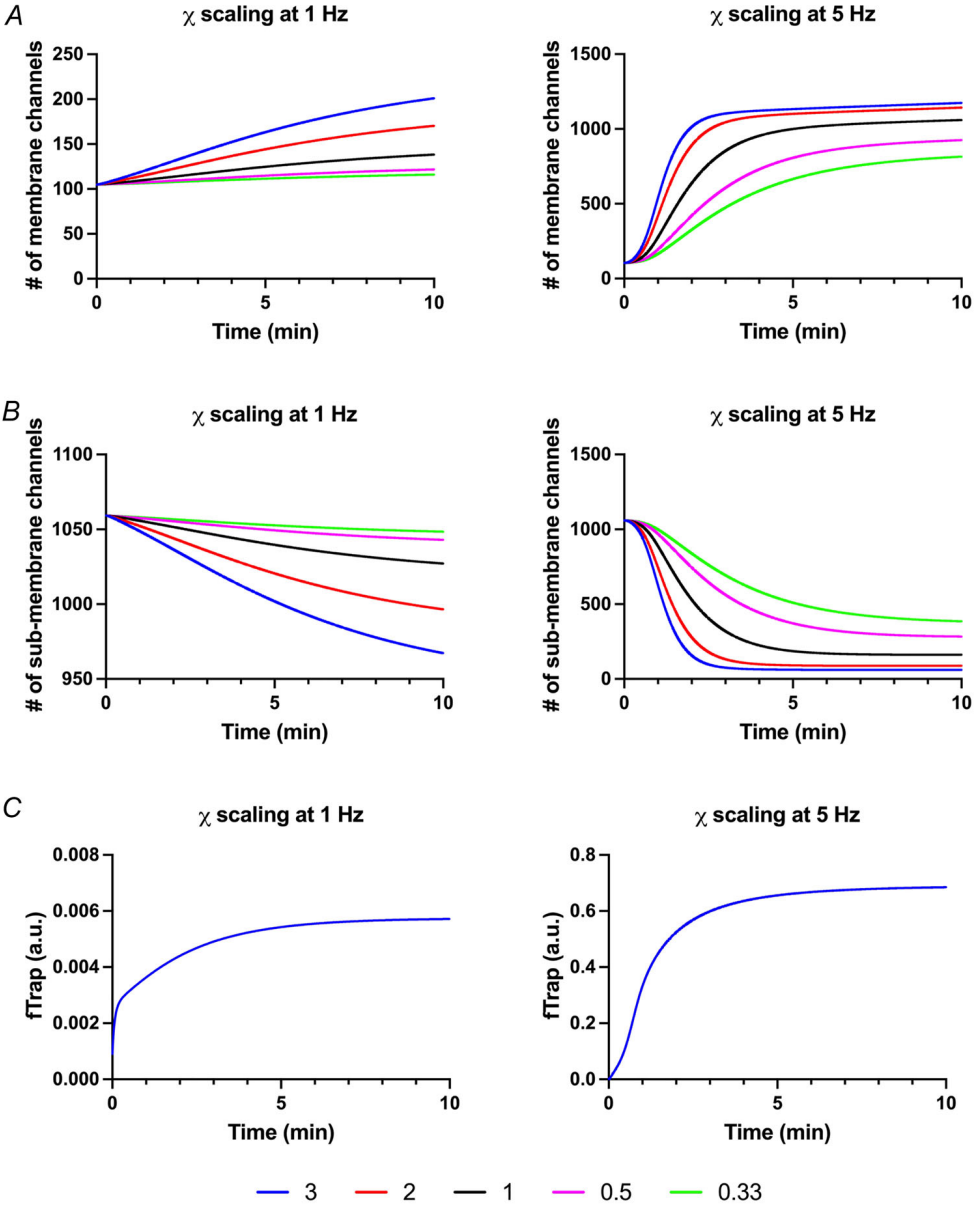
Sensitivity of SK channel behaviour to changes in the trafficking factor (χ) *A*, effect of scaling (3‐, 2‐, 1‐, 0.5‐ or 0.33‐fold) χ on the number of SK channels in the membrane and their dynamics after 10 min of triangular voltage clamp stimulation at either 1 Hz (left) or 5 Hz (right). *B*, Corresponding changes in the number of submembrane SK channels under identical conditions. *C*, Activation and dynamic behaviour of the Ca^2^⁺ sensor at different χ values, highlighting the rate‐dependent differences between 1 Hz (left) and 5 Hz (right) stimulations.

**Figure 6:**
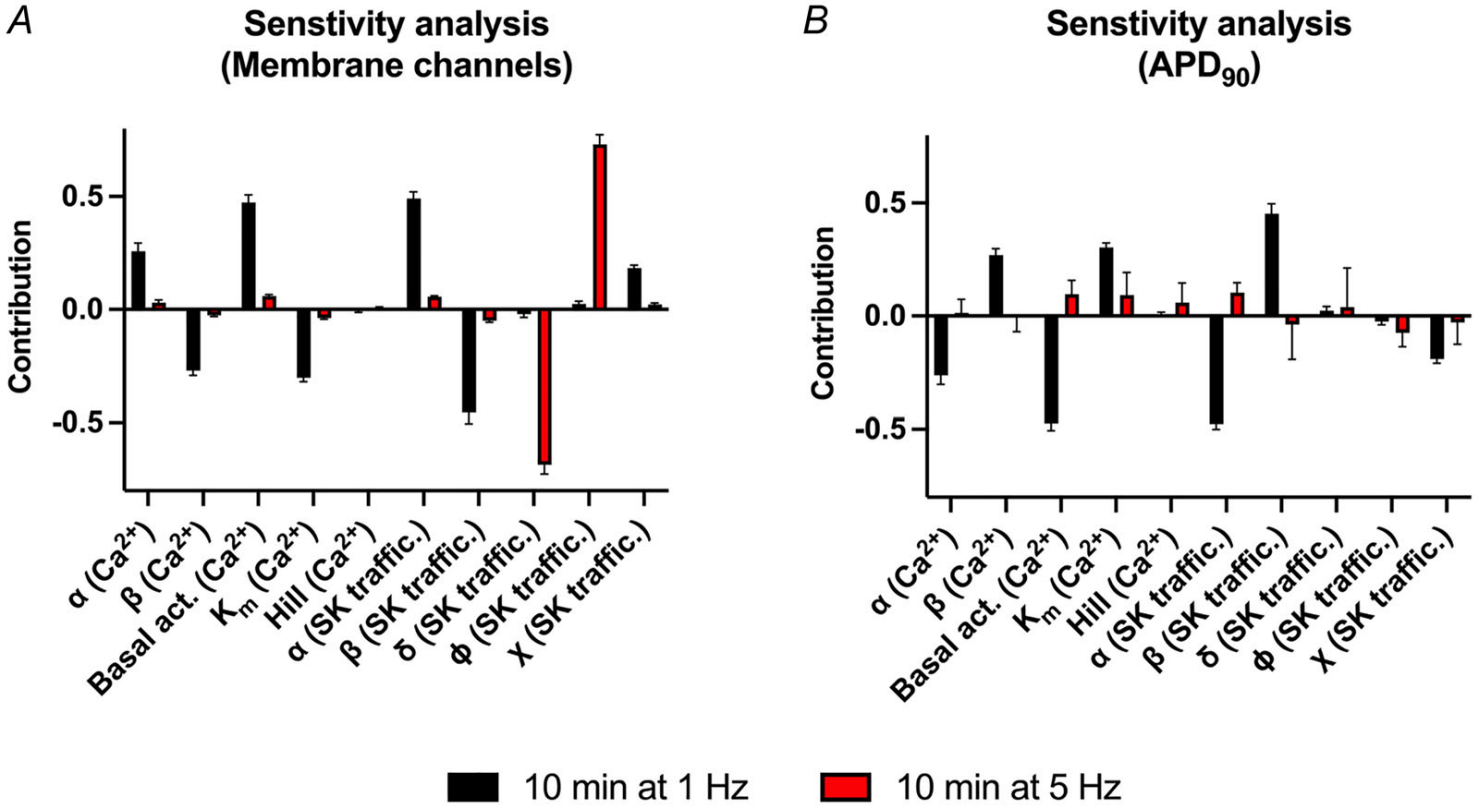
Population of models (PoM) analysis of SK trafficking and Ca^2^⁺ sensor parameters SK‐channel trafficking and Ca^2^⁺ sensor parameters were randomly varied using a normal distribution (σ = 0.1) to generate 100 distinct parameter sets. This protocol was repeated five times to evaluate the impact of each parameter on SK membrane channels (*A*) and APD (*B*) after 1 min at 1 Hz (black) followed by 10 min at 5 Hz pacing (shown in red), and to compute the mean ± SD of their influence under each condition.

A sensitivity analysis was conducted to investigate the influence of individual Ca^2^⁺‐sensor parameters on the dynamics of SK‐channel membrane levels during sinus rhythm (1 Hz) and AF‐like conditions (5 Hz). The three key parameters, namely Ca^2^⁺‐sensor activation rate (α), deactivation rate (β) and trafficking factor (χ), were scaled individually (3‐, 2‐, 1‐, 0.5‐ and 0.33‐fold), whereas the remaining parameters were held constant at their optimized values. Increasing α led to an almost linear rise in SK membrane levels at 1 Hz and a sigmoidal increase at 5 Hz, with significantly higher membrane channel levels observed at 5 Hz compared to 1 Hz (Fig. 3*A*). Higher α values also caused a leftward shift in the curve, indicating faster trafficking dynamics, particularly under 5 Hz pacing (Fig. 3*A*, right). Submembrane SK levels exhibited an inverse relationship: higher α values reduced submembrane levels as more channels were transported to the membrane (Fig. 3*B*). Furthermore, larger α values amplified Ca^2^⁺‐sensor activation, especially under high‐frequency pacing, where activation dynamics also accelerated with increased α (Fig. 3*C*). Because the Ca^2^⁺ sensor includes only two rates, scaling β produced opposite effects. Increasing β decreased SK membrane levels and slowed trafficking dynamics (Fig. 4*A*), whereas submembrane levels rose correspondingly (Fig. 4*B*). Smaller β values amplified sensor activation, with more pronounced and rapid responses at 5 Hz (Fig. 4*C*). Increasing χ led to a greater number of SK channels in the membrane (Fig. 5*A*) and fewer in the submembrane (Fig. 5*B*), with faster and more pronounced effects observed under high‐frequency stimulation. Unlike α and β, χ did not alter Ca^2^⁺‐sensor activation or deactivation because it reflects the trafficking outcome of the sensor rather than its internal dynamics (Fig. 5*C*). In addition, we implemented a population‐based modelling framework in which SK‐channel trafficking and Ca^2^⁺‐sensor parameters were randomly varied, following the approach of Heijman et al. (2023). We generated 100 distinct parameter sets and repeated the simulation protocol five times to assess the impact of each parameter on membrane channels (Fig. 6*A*) and APD (Fig. 6*B*) at both slow and fast pacing rates. At 1 Hz, membrane channel levels were primarily driven by fast trafficking kinetics and Ca^2+^‐sensor dynamics, whereas, at 5 Hz, slower processes such as protein production and degradation became the dominant contributors. During 1 Hz pacing, the relative impact of each parameter on APD parallels that of the SK‐channel levels (albeit in opposite direction because an increase in SK‐channel levels produces a reduction in APD). By contrast, during 5 Hz pacing, the absolute impact of all parameters on APD is smaller because the average APD is much shorter and the parameters controlling the Ca^2+^ sensor have the most pronounced influence, supporting an important role of Ca^2+^‐dependent SK‐channel trafficking in shaping APD during 5 Hz pacing.

### The effect of SK channels on repolarization during simulated paroxysmal AF

**Figure 7:**
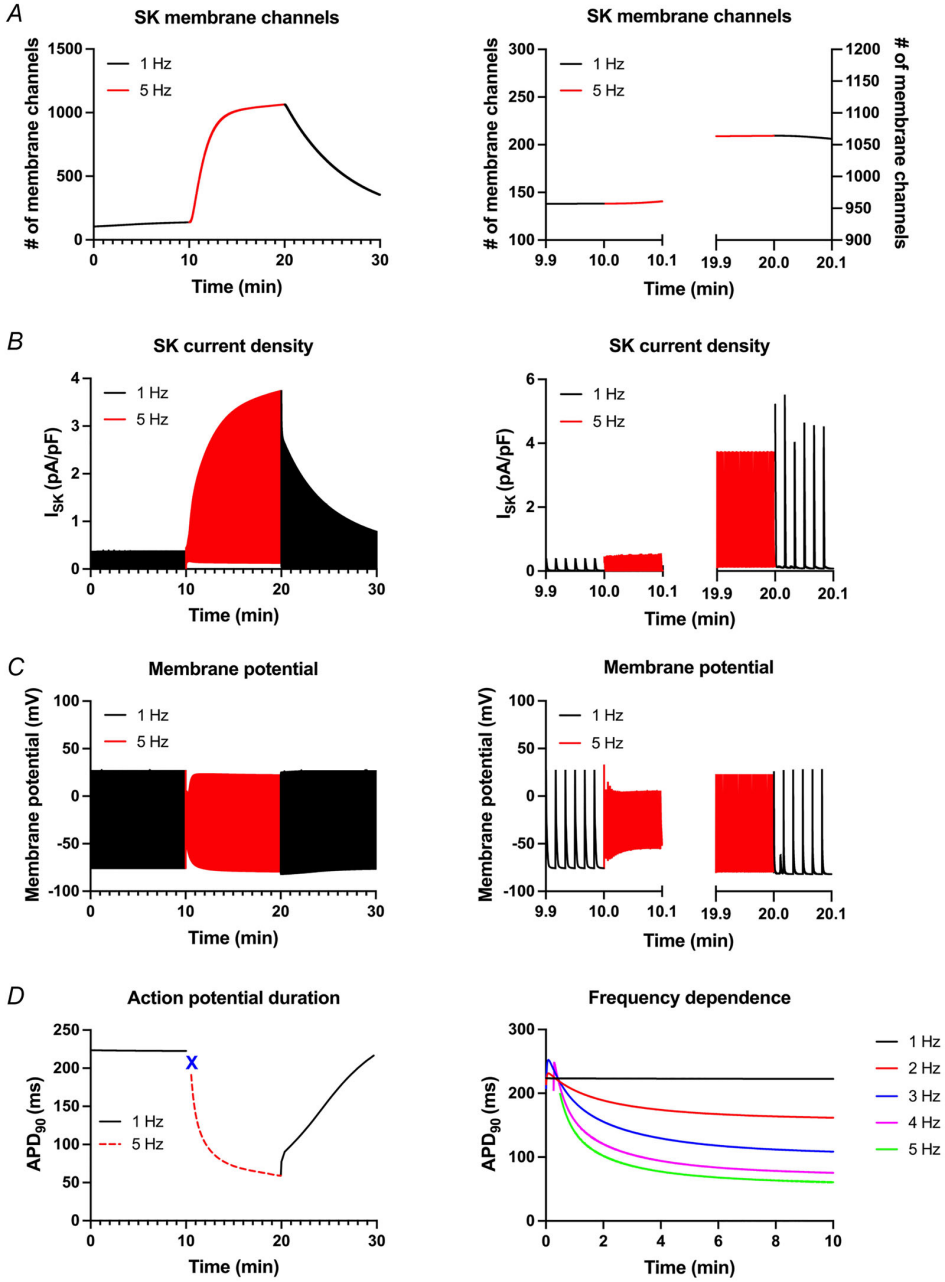
Simulated SK channel and electrophysiological responses during and after an episode of rapid activation *A* and *B*, SK‐channel membrane levels (*A*) and corresponding SK current (*I*_SK_) (*B*) during 10 min of simulated sinus rhythm simulation (1 Hz activation by triangular voltage clamp pulses), followed by 10 min at 5 Hz (mimicking AF), and a return to 1 Hz for 10 min. Right: zoomed‐in view of the transitions between frequency changes to highlight acute changes. *C* and *D*, membrane potential changes (*C*) and corresponding action potential duration (APD_90_) (*D*) during pacing in current clamp mode for the same durations as in (*A*). The blue cross highlights instances with incomplete repolarization, precluding calculation of APD_90_. *D*, right: time‐dependent changes in APD_90_ at 1, 2, 3, 4 and 5 Hz pacing rates.

Once the model was calibrated, we simulated a brief 10 min episode of paroxysmal AF to investigate the regulation of SK‐channel gating and trafficking, as well as their reversibility upon return to sinus rhythm. The simulation began with 10 min of triangular stimulation at 1 Hz to represent sinus rhythm, followed by 10 min of 5 Hz triangular stimulation to mimic an AF episode. After the AF‐like episode, 1 Hz pacing was resumed. There was an ∼8‐fold increase in SK membrane channels after 10 min of 5 Hz stimulation, and, following the return to sinus rhythm, the number of SK channels in the membrane slowly decreased (Fig. 7*A*). A similar increase (∼10‐fold) in *I*_SK_ was observed during the 5 Hz stimulation, with a gradual decline after sinus rhythm was restored (Fig. 7*B*).

Next, we investigated the effects of rate‐dependent SK‐channel remodelling on membrane potential and APD_90_ in current clamp mode. The membrane potential was stable during the first 10 min of sinus rhythm (1 Hz). When switching to 5 Hz pacing, there was a transient decrease in the peak membrane potential, accompanied by a more depolarized resting membrane potential (RMP) (Fig. 7*C*). This behaviour persisted for a brief period and precluded calculation of APD_90_ because the AP did not fully return to the resting RMP (blue cross‐marker in Fig. 7*D*, left). After this initial phase, APD_90_ declined progressively during the remainder of the 10 min of 5 Hz pacing. Following the return to sinus rhythm, APD_90_ gradually returned to baseline levels (Fig. 7*D*, left). Finally, we explored the impact of the frequency of the simulated AF‐like episode, finding that higher pacing frequencies produced a more pronounced decrease in APD_90_ over time, but that substantial APD_90_ reductions were already observed with 3 Hz pacing (Fig. 7*D*, right).

**Figure 8:**
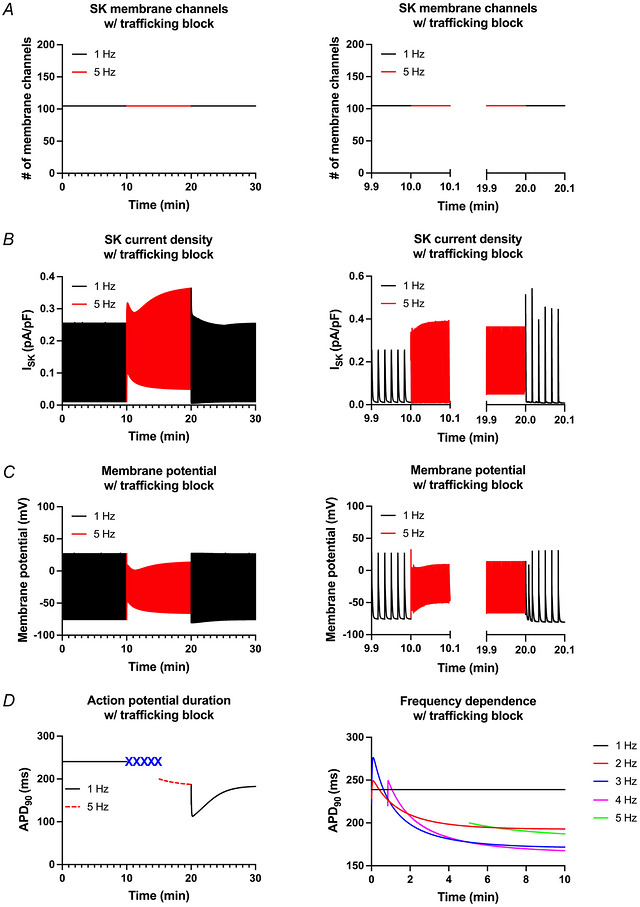
Simulated SK channel and electrophysiological responses during and after an episode of rapid activation while blocking SK‐channel trafficking *A* and *B*, SK‐channel membrane levels (*A*) and corresponding SK current (I_SK_) (*B*) during 10 min of simulated sinus rhythm simulation (1 Hz activation by triangular voltage clamp pulses), followed by 10 min at 5 Hz (mimicking AF), and a return to 1 Hz for 10 min in the absence of changes in SK‐channel trafficking. The right panel provides a zoomed‐in view of the transitions between frequency changes to highlight acute changes. *C* and *D*, membrane potential changes (*C*) and corresponding action potential duration (APD_90_) (*D*) during pacing in current clamp mode for the same durations as in (*A*). The blue cross highlights instances with incomplete repolarization, precluding calculation of APD_90_. *D*, right: time‐dependent variation in APD_90_ at 1, 2, 3, 4 and 5 Hz pacing rates.

Multiple factors, including restitution properties of ion channels and accumulation of intracellular Na^+^ and Ca^2+^, can contribute to rate‐dependent alterations in membrane potential and APD_90_ (Decker et al., 2009). To examine the role of SK‐channel regulation in this context, we repeated the previous set of simulations at the same time as completely blocking SK‐channel trafficking. By design, there was no change in SK‐channel membrane levels under these conditions (Fig. 8*A*). However, a slight increase in *I*_SK_ was observed upon switching to 5 Hz, highlighting the immediate effects of increased Ca^2^⁺ loading during AF and the corresponding increase in SK activity (Fig. 8*B*). This effect, however, was much smaller than what was observed when trafficking was enabled (Fig. 7*B*). In the absence of SK‐channel trafficking, there was a much longer period of incomplete repolarization during 5 Hz pacing (Fig. 8*C* and blue crosses in Fig. 8*D*), followed by a much smaller reduction in APD_90_ (Fig. 8*D*). The elevated RMP also reduced excitability, resulting in a couple of short APD_90_ values upon return to 1 Hz pacing. The overall rate‐dependent APD_90_ adaptation was similarly blunted for 2, 3 and 4 Hz pacing, albeit without a long period of incomplete repolarization (Fig. 8*D*, right).

### Comparison between SK‐channel block and inhibition of other K^+^ channels

To explore potential interactions between *I*_SK_ block and inhibition of other potentially relevant K^+^ currents, we performed additional cellular simulations comparing *I*_SK_ block with *I*_Kur_, *I*_Kr_, *I*_K2P_ and combined *I*_K2P_ + *I*_SK_ inhibition during 1 and 5 Hz pacing. *I*_SK_ block was implemented as an 80% reduction in current amplitude, whereas *I*_Kur_ block was implemented using the AVE0118 formulation from Grandi et al. (2011) at 10 µmol L^−1^ to capture both its concentration and frequency dependence (Grandi et al., 2011). A clinically relevant dose of dofetilide (3.8 nmol L^−1^) was used to simulate the effects of *I*_Kr_ block, resulting in a 30% reduction in *I*_Kr_ (Meier et al., 2023). Finally, *I*_K2P_ block was modelled as an 80% reduction to enable direct comparison with the degree of SK‐channel block and the previously reported synergistic effects of combined *I*_SK_ and *I*_K2P_ block (Dasí et al., 2026; Heijman et al., 2023).

**Figure 9:**
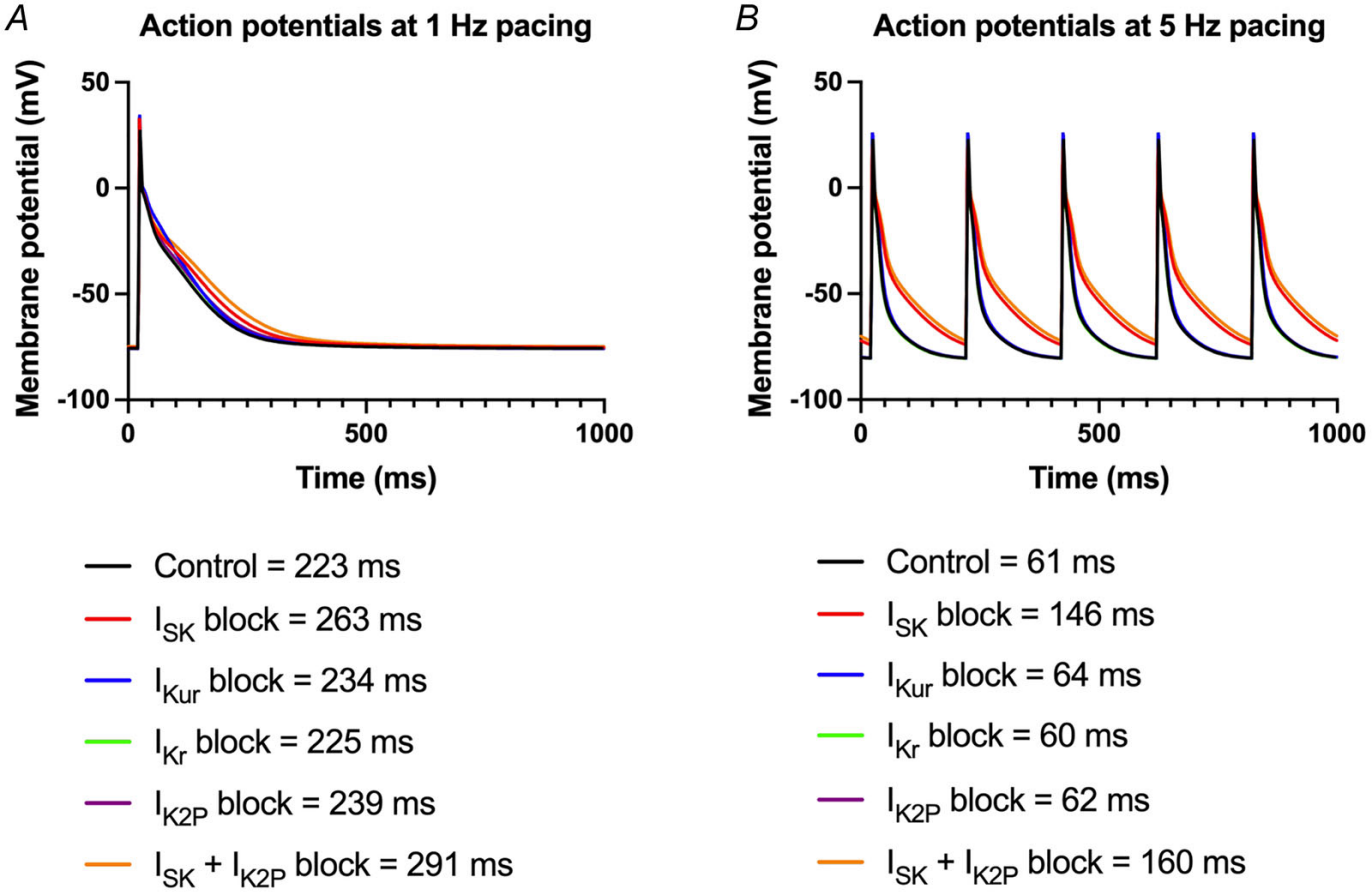
Action potentials under different drug block conditions and pacing frequencies *A*, Action potentials and corresponding action potential duration (APD_90_, shown in the legend) of the last beat during 10 min of pacing at 1 Hz under control conditions, *I*_SK_ block, *I*_Kur_ block, *I*_Kr_ block, *I*_K2P_ block or *I*_SK_ + *I*_K2P_ block. *B*, Similar to (*A*) for the last second of 5 Hz pacing for 10 min, after 10 min of initial pacing at 1 Hz, for all drug conditions.

The APD_90_ was 223 ms under control conditions after 1 Hz pacing for 10 min. In the presence of *I*_Kur_, *I*_Kr_, *I*_SK_ or *I*_K2P_ block, APD_90_ was 234, 225, 263 and 239 ms, respectively (Fig. 9*A*). The combined effects of *I*_K2P_ and *I*_SK_ block resulted in an APD_90_ of 291 ms. After an additional 10 min of 5 Hz pacing, APD_90_ was abbreviated to 61 ms in control, 64 ms with *I*_Kur_ block, 60 ms with *I*_Kr_ block, 146 ms with *I*_SK_ block, 62 ms with *I*_K2P_ block and 160 ms with combined block of *I*_SK_ and *I*_K2P_ (Fig. 9*B*). These results indicate that SK‐channel inhibition has the most pronounced APD_90_‐prolonging effect at both slow and fast pacing rates, suggesting a potentially stronger antiarrhythmic effect than *I*_Kur_ or *I*_Kr_ block. Furthermore, the combined effects of *I*_SK_ and *I*_K2P_ block resulted in a synergistic APD_90_ prolongation under both pacing conditions, consistent with previous observations (Dasí et al., 2026).

### Role of SK channels in arrhythmogenesis

**Figure 10:**
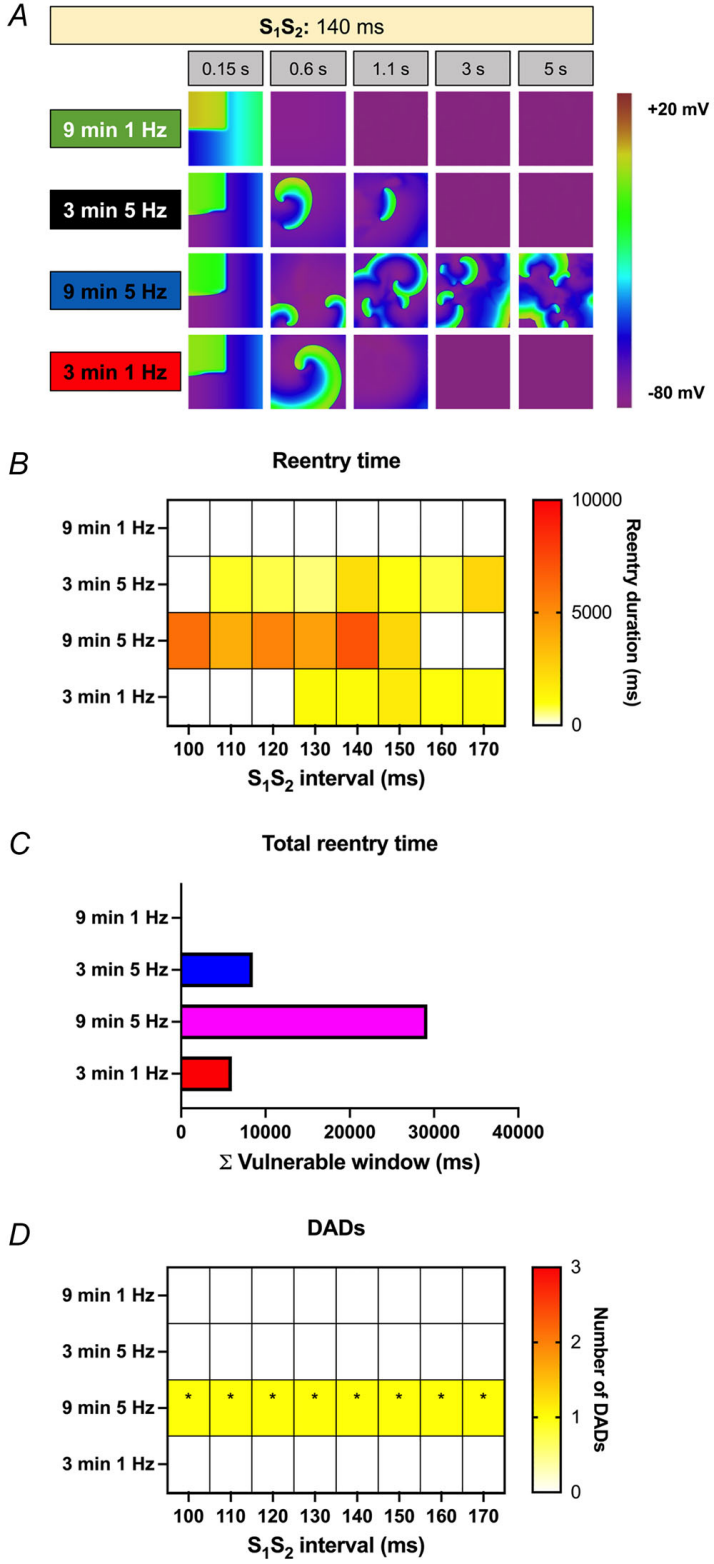
Re‐entry dynamics and arrhythmogenic risk during AF and recovery Re‐entry dynamics were assessed using 2‐D atrial‐tissue simulations (6 × 6 cm) initialized with the conditions from different durations of cellular pacing at 1 or 5 Hz in current clamp mode. *A*, representative snapshots of electrical activity for an S_1_S_2_ interval of 14 ms. Top row: based on conditions after 9 min of 1 Hz pacing, where S_2_ was blocked because of prior S_1_ activation. Second and third row: based on conditions after 3 or 9 min of 5 Hz pacing, where re‐entry was inducible, and re‐entry duration and wave break complexity increased after 9 min. Bottom row: based on conditions 3 min after resumption of 1 Hz pacing following 10 min of 5 Hz pacing; re‐entry remained inducible, but duration declined significantly compared to 9 min of 5 Hz pacing. *B*, quantification of the re‐entry durations for all S_1_S_2_ intervals for each condition. *C*, sum (Σ) of re‐entry durations for all S_1_S_2_ intervals in the four conditions, revealing a ∼4‐fold increase in total arrhythmogenic risk compared to shorter AF durations or 1 Hz pacing after 9 min of rapid activation. *D*, Number of subthreshold (*) or suprathreshold (**) DADs occurring after cessation of re‐entry in the four conditions.

Our cellular findings were further investigated in 2‐D tissue simulations to evaluate the arrhythmogenic implications of SK‐channel trafficking. The 2‐D tissue simulations showed no re‐entrant activity across all tested S_1_S_2_ intervals under baseline initial conditions (obtained after 1 Hz single cell pacing for 10 min). By contrast, when initializing the 2‐D tissue simulations with data obtained after 3 min of single‐cell pacing at 5 Hz, re‐entry was inducible with S_1_S_2_ intervals of 110–170 ms (Fig. 10*A* and *B*), resulting in a total re‐entry duration of 8505 ms (Fig. 10*C*). With the conditions after 9 min of AF‐mimicking activity, the re‐entrant S_1_S_2_ interval shifted to 100–150 ms, with more sustained re‐entries and complex wave breaks (Fig. 10*A* and *B*). The total duration of inducible re‐entry nearly tripled after 9 min compared to 3 min of 5 Hz activity, reaching 29,205 ms (Fig. 10*C*), and was accompanied by subthreshold DADs across all S_1_S_2_ intervals after re‐entry ceased (Fig. 10*D*). When the 2‐D tissue simulations were performed based on initial conditions 3 min after resuming 1 Hz pacing (mimicking conversion to sinus rhythm), the re‐entry duration reduced significantly to 6005 ms and no more subthreshold DADs were detected (Fig. 10*B*). However, re‐entry duration remained elevated compared to baseline, suggesting an increased risk for arrhythmia‐reinitiation.

**Figure 11:**
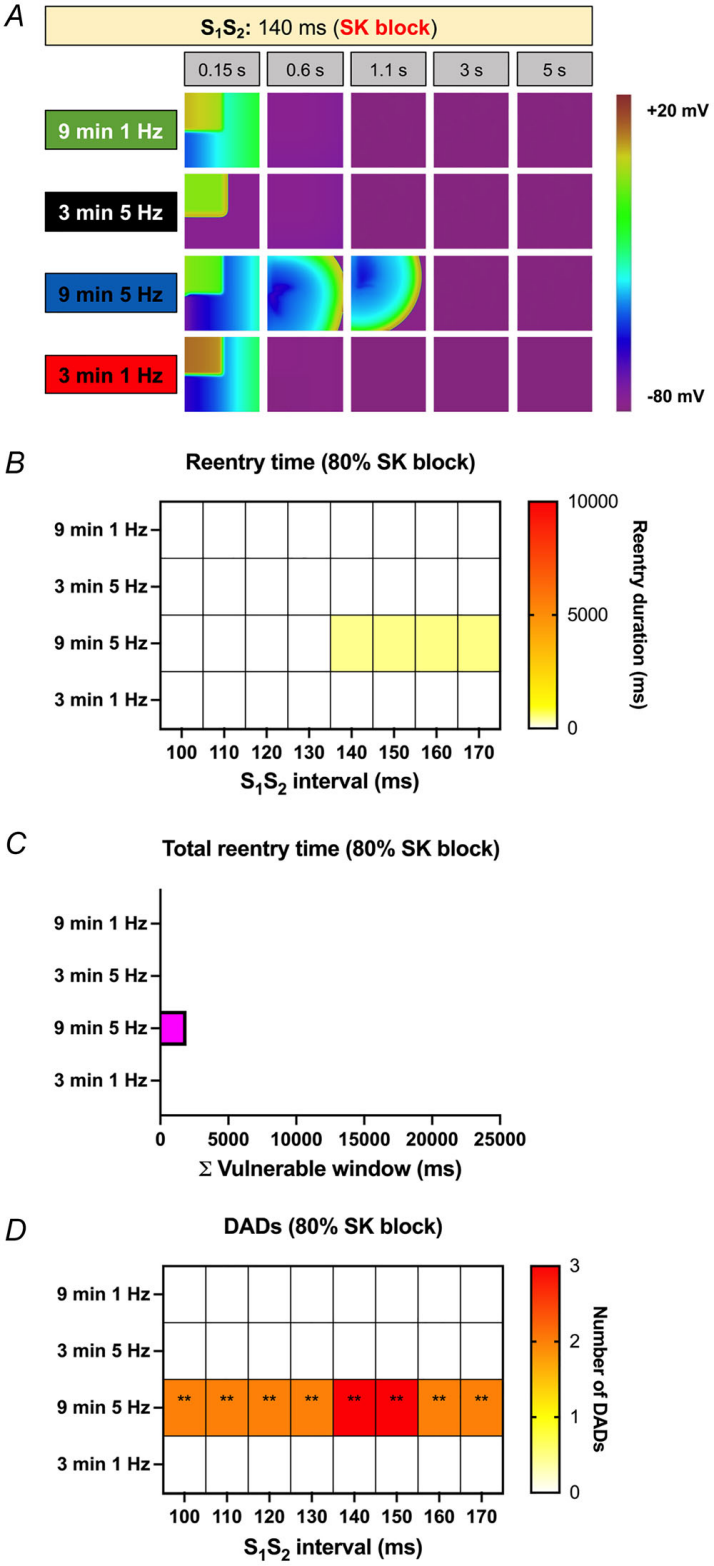
Effects of SK‐channel block on arrhythmogenic risk and re‐entry dynamics Re‐entry dynamics were assessed using 2‐D atrial‐tissue simulations (6 × 6 cm) initialized with the conditions from different durations of cellular pacing at 1 or 5 Hz in the presence of 80% SK‐channel block, in current clamp mode. *A*, representative snapshots of electrical activity for an S_1_S_2_ interval of 140 ms in the presence of 80% SK‐channel block. Top row: based on conditions after 9 min of 1 Hz pacing, where S_2_ was blocked because of prior S_1_ activation. Second and third row: based on conditions after 3 or 9 min of 5 Hz pacing; with short‐lasting re‐entry only inducible after 9 min of 5 Hz stimulation, followed by suprathreshold DADs after 0.6 and 1.1 s. Bottom row: based on conditions 3 min after resumption of 1 Hz pacing following 10 min of 5 Hz pacing; re‐entry was not inducible. *B*, quantification of the re‐entry durations for all S_1_S_2_ intervals for each condition. *C*, sum (Σ) of re‐entry durations for all S_1_S_2_ intervals in the four conditions. The total re‐entry duration was reduced by >90% to 1930 ms after 9 min of 5 Hz activity under SK‐channel block, compared to Fig. 8*C*. *D*, number of subthreshold (*) or suprathreshold (**) DADs occurring after cessation of re‐entry in the four conditions. SK‐channel block promoted suprathreshold DADs.

Next, we repeated these simulations under 80% SK‐channel block, to assess the role of SK‐channel remodelling in re‐entry promotion. With SK‐channel block, the tissue remained refractory after 3 min of 5 Hz pacing, precluding re‐entry initiation by the S_1_S_2_ stimulus. After 9 min of 5 Hz pacing with SK‐channel block total re‐entry time was significantly reduced to 1930 ms, with re‐entry confined to S_1_S_2_ intervals of 140–170 ms (Fig. 11*A*–*C*). However, two or three suprathreshold DADs (i.e. triggered APs) appeared across all S_1_S_2_ intervals after cessation of re‐entry in simulations based on 9 min of 5 Hz pacing (Fig. 11*D*). When using the initial conditions from cellular simulations after reverting to sinus rhythm (1 Hz), both re‐entries and suprathreshold DADs were eliminated within 3 min in the presence of SK‐channel block. Thus, prolonged AF pacing promotes more sustained re‐entry, whereas SK channel block diminishes re‐entry duration, but promotes the incidence of suprathreshold DADs after short paroxysmal AF‐like periods, pointing to a potential reduction of AF burden, but not elimination of AF *per se*.

## Discussion

We have developed the first computational model of SK‐channel trafficking that incorporates frequency‐dependent regulation through a novel Ca^2^⁺ sensor component. Model calibration under buffered [Ca^2+^]*_i_* conditions, closely matching experimental protocols, revealed that tachypacing increases SK‐channel trafficking and *I*_SK_, effects that were even more pronounced under free Ca^2^⁺ conditions because of direct Ca^2+^‐dependent channel gating. Our sensitivity analyses confirmed that higher stimulation frequencies mimicking AF enhanced activation of the Ca^2^⁺ sensor, resulting in increased and accelerated SK‐channel trafficking to the membrane. After 10 min of 5 Hz pacing, mimicking a paroxysmal AF episode, the APD₉₀ was significantly shortened as a result of increased *I*_SK_ and increased SK‐channel trafficking. Upon reverting to 1 Hz pacing, APD₉₀ returned to normal levels, reflecting a reduction in SK channel density at the membrane. Initializing 2‐D tissue simulations based on AF‐like conditions (5 Hz), progressively prolonged re‐entry persistence and promoted ectopic (triggered) activity. SK‐channel blockade shortened re‐entry duration but paradoxically increased ectopic activity, revealing the potential contribution of SK channels to AF‐maintaining re‐entry and AF progression, at the same time as suppressing triggered activity.

### The role of Ca^2+^ in SK‐channel functioning and trafficking

Intracellular Ca^2+^ plays a dual role in SK‐channel regulation, mediating both channel gating and trafficking. Gating is initiated by Ca^2^⁺ binding to CaM, which is tightly associated with SK‐channels, leading to channel opening and subsequent deactivation upon Ca^2^⁺ dissociation. Beyond gating, [Ca^2+^]*_i_* influences the trafficking and surface expression (targeting) of SK‐channels (Heijman et al., 2023; Saljic et al., 2024). This dynamic regulation underscores the complexity of mimicking Ca^2^⁺‐mediated effects in computational models. To replicate the experimental conditions of Heijman et al. (2023), where [Ca^2+^]*_i_* was fixed at 500 nmol L^−1^ using EGTA, we incorporated similar buffering in our simulations. The slow binding kinetics of EGTA result in spatially restricted buffering, allowing localized changes in [Ca^2+^]*_i_* proximal to LTCC and the activation of nearby ryanodine receptors (RyR2) (Eisner et al., 2023; Kornyeyev et al., 2010). To mimic this effect of EGTA in our model, we only fixed the cytosolic (but not subsarcolemmal or subspace) Ca^2+^ concentration. However, more advanced methods incorporate EGTA's binding kinetics and spatial compartmentalization, making them more physiologically accurate (Kornyeyev et al., 2010). Although our approach simplifies the detailed dynamics of EGTA‐mediated Ca^2+^ buffering, it successfully maintains [Ca^2+^]*_i_* at a stable level through the simulations and the model could accurately simulate the observed experimental behaviour (Heijman et al., 2023). Nonetheless, spatial coupling was implicitly considered because the Ca^2^⁺ sensor and SK‐channel forward trafficking were controlled by subsarcolemmal Ca^2^⁺, which is modulated by LTCC and RyR2 activity. This connection to LTCCs is supported by the nifedipine simulations, which show a similar reduction in SK‐channel trafficking and gating as observed experimentally (Heijman et al., 2023). Although phenomenological, this approach can be refined as more experimental data on Ca^2^⁺‐dependent regulation and SK‐channel localization in human atrial cardiomyocytes become available. After calibrating the model, simulations with free intracellular Ca^2+^ (i.e. without demoting the cytosolic Ca^2+^ equation, producing normal Ca^2+^ transients) predicted an even greater increase in SK‐channel trafficking and larger *I*_SK_ after tachypacing. Although these results could not be validated directly because of the lack of experimental data, they are consistent with the buffering effect of EGTA limiting the increase in [Ca^2+^]*_i_* at fast rates and the combined promotion of SK‐channel gating and trafficking by Ca^2+^.

### Tachypacing as a modulator of SK functioning and trafficking

The majority of data on pacing‐induced upregulation of SK‐channel activity comes from large animal models where rapid atrial pacing was performed for days. For example, Qi et al. (2014) performed atrial tachypacing at ∼7 Hz for 7 days in dogs, showing increased SK‐channel activity compared to control dogs, although this increase was attributed to increased open probability of the channels. Several studies have also investigated pacing‐dependent SK‐channel regulation over shorter timeframes (hours instead of days). Rahm et al. (2021) identified a reduction in SK3 protein levels and unchanged total levels of SK1 and SK2 after 24 h of 5 Hz tachypacing of HL‐1 cells but trafficking or SK‐channel function was not assessed. On the other hand, Li et al. (2018) showed that SK2 membrane trafficking was significantly upregulated after 24 h of 10 Hz tachypacing in neonatal rat ventricular myocytes. Similarly, Özgen et al. (2007) performed intermittent rapid atrial burst‐pacing in rabbits for 3 h, showing increased SK‐channel trafficking and expression, resulting in more membrane channels, increased *I*_SK_ and shortened APD after rapid pacing. Of note, the effective pacing time in this study was only 18 min of the entire protocol. Together, the studies by Li et al. (2018) and Özgen et al. (2007) indicated that SK‐channel trafficking is upregulated in response to tachypacing of isolated cardiomyocytes over much shorter timeframes (i.e. minutes) than previously investigated (i.e. hours/days). This hypothesis was subsequently tested by Heijman et al. (2023), who revealed that SK‐channel trafficking plays a crucial role in the upregulation of *I*_SK_ in atrial cardiomyocytes from patients with persistent AF. They observed an increased number of SK2 channels in the membranes of AF cardiomyocytes compared to controls. When anterograde trafficking was inhibited with latrunculin‐A or retrograde trafficking with primaquine, this upregulation was reversed, returning to control levels (Heijman et al., 2023). Furthermore, SK‐channel trafficking proved highly dynamic, as just 10 min of 5 Hz stimulation significantly increased membrane SK‐channel density and *I*_SK_ to levels observed in AF cardiomyocytes.

These findings highlight how short periods of tachypacing can enhance SK‐channel trafficking and *I*_SK_. Fast trafficking dynamics have also been observed for other cardiac ion channels, such as K_V_11.1, which underlies the rapidly activating delayed‐rectifier K^+^ current (*I*_Kr_). A high‐throughput flow cytometry study has reported K_V_11.1 channel trafficking on the order of minutes (Kanner et al., 2018), which is in striking contrast to earlier studies using techniques such as an enzyme‐linked immunosorbent assay and western blotting, which suggested timescales of hours (Meier et al., 2023). Additionally, two studies demonstrated K_V_11.1 channel internalization and recycling processes completing within minutes, with these findings initially being puzzling, whereas they are now in alignment with the broader evidence of rapid trafficking dynamics (Apaja et al., 2013; Dennis et al., 2011). Similarly, Li et al. (2018) used flow cytometry to measure SK‐channel internalization half‐times of ∼30 min. These observations highlight the critical role of employing high‐resolution techniques to accurately capture ion channel trafficking dynamics (Meier et al., 2023).

Moreover, our results suggest that Ca^2+^‐dependent promotion of SK‐channel trafficking already occurs at 4 Hz, 3 Hz and, to a lesser degree, also at 2 Hz. To the best of our knowledge, experimental data are unavailable to validate our model's frequency‐dependent Ca^2+^ sensor for slower stimulation frequencies (i.e. 2–4 Hz) over short timeframes (i.e. 10 min). However, if these data are confirmed in future experimental studies, this could also have significant implications for understanding exercise‐related AF. For example, an elevated heart rate during a brief period of exercise could then already increase SK‐channel trafficking to the membrane, potentially exacerbating the effects of the exercise‐related sympathovagal activation (Gorman et al., 2024) and promoting AF induction shortly after cessation of exercise in the presence of a sufficiently vulnerable substrate.

### SK channels as novel antiarrhythmic targets

Extensive *in vivo*, *ex vivo* and clinical studies from the last 20 years have positioned SK channels as promising targets for novel therapeutic strategies for rhythm control of AF (Saljic et al., 2024). Early research in rodents (e.g. rats and guinea‐pigs) and large animal models (e.g. dogs, pigs and horses) demonstrated that different SK‐channel inhibitors and modulators, such as NS8593, UCL1684 and AP14145, significantly prolonged the atrial effective refractory period, reduced AF vulnerability and prevented AF reinduction, with few unwanted ventricular or haemodynamic effects (Saljic et al., 2024). Building on these preclinical data, AP30663 became the first SK‐channel blocker to undergo clinical testing. In a phase II clinical trial, AP30663 demonstrated efficacy in converting recent‐onset AF to sinus rhythm, achieving cardioversion rates comparable to vernakalant and flecainide (Saljic et al., 2024). This represents a key milestone in developing SK‐channel inhibitors for acute rhythm control. Currently, a second‐generation oral SK‐channel blocker, AP31969, is in phase I clinical trials for sinus rhythm maintenance (Saljic et al., 2024). These developments underscore the promise of SK channel inhibitors as both acute and long‐term antiarrhythmic therapies. A recent *in silico* study by Dasí et al. (2026) demonstrated that *I*_SK_ and *I*_K2P_ block alone cardioverted 33% and 43% of virtual AF patients, respectively, whereas their combined application increased cardioversion rates to 82%. Our cellular simulations similarly showed a greater‐than‐additive APD_90_ prolongation with combined *I*_SK_ and *I*_K2P_ block, particularly under AF‐mimicking conditions. Together, these findings suggest that a multitarget pharmacological approach involving SK‐channel inhibition may represent a promising strategy for rhythm control in AF. Such an approach may also limit the ectopic activity‐promoting effects of SK‐channel inhibition that we have identified in our simulations (e.g. via concomitant inhibition of Na^+^ channels). At the same time, SK channel inhibition has been suggested to have proarrhythmic effects. Indeed, increased *I*_SK_ also augments repolarization reserve, reducing the likelihood of early afterdepolarizations, and stabilizes the RMP, which counteracts DADs, thereby protecting against atrial ectopic activity (Herrera et al., 2023). As such, SK‐channel inhibition can have both antiarrhythmic and proarrhythmic effects. The antiarrhythmic effects include prolongation of APD and refractory period, which counteracts re‐entry. However, Herrera et al. (2023) also showed that SK channel block increases vulnerability to DADs in virtual human atrial cardiomyocyte models. Our simulations are consistent with this dual effect and expand these findings to multicellular simulations, with SK‐channel block reducing re‐entry duration, but increasing the likelihood of ectopic activity after cardioversion. These findings suggest that inhibiting SK channels could either aid or hinder rhythm control in AF, depending on the dominant arrhythmia mechanism in individual patients, which together with the complexity and rate‐dependent nature of SK‐channel remodeling, makes understanding its precise impact on atrial electrophysiology challenging. Nevertheless, the net effect of inhibiting re‐entry while promoting triggered activity would be consistent with a reduction in AF burden by decreasing AF‐episode duration. Although this approach may not eliminate AF entirely, AF burden correlates with stroke risk, and reducing AF burden has been shown to lower mortality in patients with terminal heart failure (Becher et al., 2024; Sciacca et al., 2024; Sohns et al., 2021). If similar benefits extend to other AF populations, SK inhibition could represent a valuable approach to rhythm control. Future studies should investigate this potential.

### Potential limitations

Although the 2‐D tissue simulations produced results consistent with the cellular data, several limitations should be noted. First, because of computational constraints, we were unable to simulate prolonged pacing (i.e. for 10 min) in the 2‐D tissue model, and instead used snapshots of cellular pacing. This limitation may impact the accuracy of long‐term behaviour under sustained pacing. Additionally, the 2‐D tissue model used in the present study was homogeneous, whereas real cardiac tissue exhibits significant physiological heterogeneity, which could influence the patterns of electrical activation. In particular, patchy distributions of conductivity or excitability, arising from fibrosis, ion channel remodeling, or cell‐cell uncoupling, can anchor reentrant circuits or promote wavebreak. These phenomena are not captured in our homogeneous model. Such factors may affect the generalizability of our findings to more complex, realistic scenarios. Moreover, the 5 Hz pacing frequency used in our simulations is relatively slow for AF, which has dominant frequencies of >6 Hz (Sanders et al., 2005), suggesting that the effects observed in AF could be even more pronounced under faster pacing conditions. Furthermore, we employed a population of models approach to assess the influence of individual model parameters on APD. However, predicting the impact of these findings on arrhythmogenesis is challenging. For example, a redistribution of channels from the submembrane to the plasma membrane state may increase re‐entry vulnerability at baseline by causing APD abbreviation, but would reduce the proarrhythmic consequences of tachycardia‐induced trafficking promotion. Finally, we were unable to calibrate the model over a wider range of timescales (minutes to hours) and pacing frequencies as a result of a paucity of experimental data.

### Conclusions

In conclusion, we have developed the first *in silico* model that simulates the effects of tachypacing‐induced changes in SK‐channel trafficking and gating across short (milliseconds) to medium (minutes) timescales. This novel computational framework integrates rate‐dependent SK‐channel trafficking into a human atrial cardiomyocyte model, allowing us to explore the dynamic effects of SK‐channel regulation on atrial electrophysiology. Our findings highlight the potential role of SK‐channel upregulation in the early stages of AF, demonstrating that increased SK channel density and enhanced SK current contribute to shortened APD, promoting re‐entry but counteracting ectopic activity under tachypacing conditions. Although this model does not fully resolve the complex AF‐begets‐AF phenomenon, it represents an important step forward in understanding the mechanistic underpinnings of AF stabilization and provides a foundation for studying other ion‐channel dynamics, optimizing experimental protocols and generating new hypotheses to guide the development of targeted therapies for AF management. Supporting Information.

## Additional information

## Competing interests

The authors declare that they have no competing interests.

## Author contributions

All authors have approved the final version of the manuscript submitted for publication. All persons designated as authors qualify for authorship, and all those who qualify for authorship are listed.

## Funding

This work was supported by the Netherlands Organization for Scientific Research (NWO/ZonMW Vidi 09150171910029 to JH), the Deutsche Forschungsgemeinschaft (Research Training Group 2989, project 517043330 to DD), National Institutes of Health (RO1HL131517, RO1HL136389, RO1HL163277, RO1HL160992, RO1HL165704, RO1HL164838, and RO1HL176651 to DD), the European Union (large‐scale network project MAESTRIA No. 965286 to DD) and the Dutch Heart Foundation (grant number 01‐002‐2022‐0118, EmbRACE consortium to JH).

## Supporting information




Peer Review History

Statistical Summary Document

## Data Availability

The model code, optimization scripts and data are available online (https://github.com/HeijmanLab).
