# Supplementary material for: Computational modelling of the pro‐ and antiarrhythmic effects of atrial high rate‐dependent trafficking of small‐conductance calcium‐activated potassium channels
Source: J Physiol. 2025 Jul 20;604(13):5355–77. doi: 10.1113/JP288659 (PMC12326020; doi:10.1113/JP288659)
Supplement: Supplementary file 2 — Statistical Summary Document [file TJP-604-5355-s001.docx]

**Manuscript Title**: Computational Modeling of the Pro- and Antiarrhythmic Effects of Atrial High Rate-Dependent Trafficking of Small-Conductance Calcium-Activated Potassium Channels

**Authors:** Stefan **Meier**, Dobromir **Dobrev**, Paul G.A. **Volders**, and Jordi **Heijman**

**Animal model used, if applicable:** N.A.

**Underlying Hypothesis:**We hypothesized that regulation of SK channel trafficking by high atrial rates modulates cardiac electrophysiology over minutes, and that SK channel block can exert antiarrhythmic effects. This hypothesis is explored using an in-silico model of the human atrial cardiomyocyte.

**Definitions of ‘n’:**Not applicable, as we primarily used deterministic simulations, which inherently produce the same result for each simulation.

**Statistical Summary Table:**Not applicable, as this study investigates the effects of SK-channel trafficking on atrial repolarization using an in-silico model, which is not subject to measurement error.
