# Supplementary material for: Computational modelling of the pro‐ and antiarrhythmic effects of atrial high rate‐dependent trafficking of small‐conductance calcium‐activated potassium channels
Source: J Physiol. 2025 Jul 20;604(13):5355–77. doi: 10.1113/JP288659 (PMC12326020; doi:10.1113/JP288659)
Supplement: Supplementary file 1 — Peer Review History [file TJP-604-5355-s002.pdf]

The Journal of Physiology

Computational Modeling of Pro- and Antiarrhythmic Effects of Atrial High Rate-Dependent Trafficking of Small-Conductance Ca^{2+}-Activated K^+ Channels

Stefan Meier, Dobromir Dobrev, Paul GA Volders, and Jordi Heijman
DOI: 10.1113/JP288659

Corresponding author(s): Jordi Heijman (jordi.heijman@medunigraz.at)

The referees have opted to remain anonymous.

Review Timeline:

Submission Date:	30-Jan-2025
Editorial Decision:	31-Mar-2025
Revision Received:	04-Jun-2025
Accepted:	23-Jun-2025

Senior Editor: Bjorn Knollmann

Reviewing Editor: Axel Loewe

Transaction Report:

(Note: With the exception of the correction of typographical or spelling errors that could be a source of ambiguity, letters and reports are not edited. Depending on transfer agreements, referee reports obtained elsewhere may or may not be included in this compilation. Referee reports are anonymous unless the Referee chooses to sign their reports.)

Dear Dr Heijman,

Re: JP-RP-2025-288659 "**Computational Modeling of Pro- and Antiarrhythmic Effects of Atrial High Rate-Dependent Trafficking of Small-Conductance Ca2+-Activated K+ Channels**" by Stefan Meier, Dobromir Dobrev, Paul GA Volders, and Jordi Heijman

Thank you for submitting your manuscript to The Journal of Physiology. It has been assessed by a Reviewing Editor and by 2 expert referees and we are pleased to tell you that it is potentially acceptable for publication following satisfactory major revision.

Please advise your co-authors of this decision as soon as possible.

The referee reports are copied at the end of this email.

Please address all the points raised and incorporate all requested revisions or explain in your Response to Referees why a change has not been made. We hope you will find the comments helpful and that you will be able to return your revised manuscript within 2 months. If you require longer than this, please contact journal staff: jp@physoc.org. Please note that this letter does not constitute a guarantee for acceptance of your revised manuscript.

Your revised manuscript should be submitted online using the link in your Author Tasks: Link Not Available. This link is accessible via your account as Corresponding Author; it is not available to your co-authors. If this presents a problem, please contact journal staff (jp@physoc.org). Image files from the previous version are retained on the system. Please ensure you replace or remove any files that are being revised.

If you do not wish to submit a revised version of your manuscript, you must inform our journal staff (jp@physoc.org) or reply to this email to request withdrawal. Please note that a manuscript must be formally withdrawn from the peer review process at one journal before it may be submitted to another journal.

TRANSPARENT PEER REVIEW POLICY: To improve the transparency of its peer review process, The Journal of Physiology publishes online as supporting information the peer review history of all articles accepted for publication. Readers will have access to decision letters, including Editors' comments and referee reports, for each version of the manuscript, as well as any author responses to peer review comments. Referees can decide whether or not they wish to be named on the peer review history document.

ABSTRACT FIGURES: Authors are expected to use The Journal's premium BioRender account to create/redraw their Abstract Figures. Information on how to access this account is here: https://physoc.onlinelibrary.wiley.com/journal/14697793/biorender-access.

This will enable Authors to create and download high-resolution figures. If authors have used the free BioRender service, they can use the instructions provided in the link above to download a high-resolution version suitable for publication.

The link provided should only be used for the purposes of this submission. Authors will be charged for figures created on this account if they are not related to this manuscript submission.

LANGUAGE EDITING AND SUPPORT FOR PUBLICATION: If you would like help with English language editing, or other article preparation support, Wiley Editing Services offers expert help, including English Language Editing, as well as translation, manuscript formatting, and figure formatting at www.wileyauthors.com/eeo/preparation. You can also find resources for Preparing Your Article for general guidance about writing and preparing your manuscript at www.wileyauthors.com/eeo/prepresources.

RIGOUR AND REPRODUCIBILITY: We recommend authors consult The Journal's Rigour and Reproducibility requirements to ensure that their manuscript is suitable for publication: https://physoc.onlinelibrary.wiley.com/pb-assets/hub-assets/physoc/documents/TJP-Rigour-and-Reproducibility-Requirements.pdf

REVISION CHECKLIST:

Check that your Methods section conforms to journal policy: https://jp.msubmit.net/cgi-bin/main.plex?form_type=display_requirements#methods.

Check that data presented conforms to the statistics policy: https://jp.msubmit.net/cgi-bin/main.plex?form_type=display_requirements#statistics.

Upload a full Response to Referees file. To create your 'Response to Referees': copy all the reports, including any comments from the Senior and Reviewing Editors, into a Microsoft Word, or similar, file and respond to each point, using font or background colour to distinguish comments and responses and upload as the required file type.

Please upload two versions of your manuscript text: one with all relevant changes highlighted and one clean version with no changes tracked. The manuscript file should include all tables and figure legends, but each figure/graph should be uploaded as separate, high-resolution files.

You may also upload:

- 'Potential Cover Art' for consideration as the issue's cover image
- Appropriate Supporting Information (Video, audio or data set: see https://jp.msubmit.net/cgi-bin/main.plex?form_type=display_requirements#supp).

We look forward to receiving your revised submission.

If you have any queries, please reply to this email and we will be pleased to advise.

Yours sincerely,

Bjorn Knollmann
Senior Editor
The Journal of Physiology

REQUIRED ITEMS

- Author photo and profile. First or joint first authors are asked to provide a short biography (no more than 100 words for one author or 150 words in total for joint first authors) and a portrait photograph. These should be uploaded and clearly labelled together in a Word document with the revised version of the manuscript. See Information for Authors for further details.

- Please upload separate high-quality figure files via the submission form.

- Please include an Abstract Figure file, as well as the Figure Legend text within the main article file. The Abstract Figure is a piece of artwork designed to give readers an immediate understanding of the research and should summarise the main conclusions. If possible, the image should be easily 'readable' from left to right or top to bottom. It should show the physiological relevance of the manuscript so readers can assess the importance and content of its findings. Abstract Figures should not merely recapitulate other figures in the manuscript. Please try to keep the diagram as simple as possible and without superfluous information that may distract from the main conclusion(s). Abstract Figures must be provided by authors no later than the revised manuscript stage and should be uploaded as a separate file during online submission labelled as File Type 'Abstract Figure'. Please also ensure that you include the figure legend in the main article file. All Abstract Figures should be created using BioRender. Authors should use The Journal's premium BioRender account to export high-resolution images. Details on how to use and access the premium account are included as part of this email.

EDITOR COMMENTS

Reviewing Editor:

Your manuscript was reviewed by two expert referees who acknowledge that this is an interesting study addressing a relevant question. In their reports, both referees point out a number of points that need to be addressed for further consideration of this manuscript.

In addition to the reviewers' comments, I suggest:

- reducing the number of digits of values of model parameters; if the 16th digit after the point would make a significant qualitative difference, I'd be concerned about the numerical reproducibility of the results

- making a concrete version of the code available in a persistent and citable way, I could not clearly identify the relevant code from the given link. GitHub/Zenodo integration is a convenient way of doing this:
https://docs.github.com/en/repositories/archiving-a-github-repository/referencing-and-citing-content

Please also see 'Required Items' above.

Senior Editor:

I concur with the reviewing editor.

REFEREE COMMENTS

Referee #1:

Computational Modeling of the Pro- and Antiarrhythmic Effects of Atrial High Rate-Dependent Trafficking of Small-Conductance Calcium-Activated Potassium Channels

General comments:

The authors aim to address the pro- and antiarrhythmic activities of SK channels using computational studies due to the technical challenges associated with studying SK channel through electrophysiological experiments. By incorporating the SK trafficking component into a human atrial cardiomyocyte model incorporating a Ca^{2+} sensor component, they seek to gain a deeper understanding of how SK channel dysregulation contributes to arrhythmogenesis. The authors found that tachypacing in a single-cell model increased SK channel density, which in turn shortened the APD. In a 2D model, tachycardia increased reentry and ectopic activity. Furthermore, the results demonstrated that inhibiting SK currents reduced reentry but promoted ectopic activity. Given the potential of SK channels in treating cardiac arrhythmias, especially with a SK channel inhibitor currently undergoing clinical trials for the treatment of atrial fibrillation, this study holds significant clinical relevance. However, the reviewer has the following concerns regarding the manuscript.

1. Previous publications have shown that intracellular Ca plays dual roles in the SK channel regulation. Rapid pacing promotes SK channel membrane-localization, therefore this model studied a physiologically relevant question. However, both the cardiomyocyte and the SK trafficking component are based on previously reported models, the reviewer is therefore concerned of the limited novelty of this work.

2. SK channel activation depends on the increase in intracellular Ca2+ of the cardiomyocytes on a beat-to-beat basis. However, the Ca2+ handling is oversimplified in this model with a fixed subsarcolemmal Ca2+ concentration. The Ca2+-sensor of SK channels, which regulates the SK trafficking and activation, likely senses local Ca2+ and may be altered at higher frequency. Therefore, the model may not be able to predict SK channel function accurately. The author should discuss how the fixed cytosolic Ca2+ concentration affect the Ca2+-sensor function and consider incorporating more detailed Ca2+ buffering kinetics into the model.

3. SK channels have previously been shown to be coupled to L-type Ca channel (LTCC) and ryanodine receptors (RyR). The authors should consider integrating LTCC and RyR coupling into the model.

4. Calcium affinity and Hill coefficients are also key parameters of the channel trafficking, the author should perform sensitivity analysis on these parameters.

5. The 2D model provides valuable insights into the role SK channels in arrhythmogenesis, however, the model assumed a homogenous tissue structure, which could affect the accuracy of reentry.

Referee #3:

The manuscript by Meier and colleagues provides a valuable contribution by modeling how rate-dependent changes can impact the trafficking and function of small-conductance calcium-activated potassium (ISK) channels. Notably, the authors effectively illustrate the pro-arrhythmic implications of rapid pacing and assess the therapeutic potential of ISK inhibition in 2D-tissue simulations. Integrating calcium-sensitive channel trafficking into a human atrial cardiomyocyte model offers a novel and temporally dynamic perspective on membrane conductance. This could be particularly important for the patient-centered management of atrial fibrillation (AF).

Suggestions

-The model presents significant strengths, particularly its multi-scale development and detailed exploration of ISK channel dynamics in the context of AF. To build further upon this solid foundation, the authors might consider extending their modeling approach to explore how variations in other clinically relevant ionic currents, targeted by existing AF therapies, can influence the outcomes observed with ISK modulation/2D simulation. For instance, evaluating the interplay between ISK inhibition and commonly used anti-arrhythmic drugs could provide insights into synergistic or additive therapeutic effects, potentially uncovering novel benefits or limitations of current treatments.

-A population-based modeling framework could substantially enrich the robustness and translational potential of key findings. By exploring variability across simulated patient populations, the authors could identify factors that enhance or diminish vulnerability to AF related to ISK trafficking dynamics. Such an approach would also help pinpoint conditions (such as specific disease states or genetic variants) that may uniquely influence ISK channel trafficking and thus modify arrhythmic risk.

Overall, the current manuscript provides important foundational knowledge and conceptual innovation. Considering some of these suggestions or applying the model in another way to generate new knowledge would improve the manuscript's impact by providing insights into AF mechanisms and therapy. These simulations could yield new insights into different therapeutic strategies/risks, thereby significantly enhancing the clinical implications of this important model.

END OF COMMENTS

Response to Reviewer Comments - JP-RP-2025-288659

We thank the Editor and the Reviewers for their thorough assessment of our manuscript and their thoughtful suggestions for further improvement. We have provided a point-by-point response to all comments below.

Editor comments:

Comment: Reducing the number of digits of values of model parameters; if the 16th digit after the point would make a significant qualitative difference, I'd be concerned about the numerical reproducibility of the results.

Response: We thank the editor for this helpful suggestion. We have revised the parameter tables to maintain a consistent level of significance, ensuring that all parameter values are presented with three significant digits to enhance numerical readability and reproducibility.

Table 1. SK-channel trafficking parameters. Parameters controlling changes in SK channel numbers are based on a timescale of hours.

**Parameter**	**Value**
α	8.20×10^{-1}
β	8.29
δ	4.80×10^{-1}
ψ	5.08×10^{2}
χ	1.00×10^{2}

Table 2. Ca^{2+} sensor parameters. Parameters controlling changes in Ca^{2+} sensor activity are based on a timescale of milliseconds, where applicable.

**Parameter**	**Value**
a	5.00×10^{-2}
b	3.50×10^{-4}
K_m	1.15×10^{-3}
h	4.00

Table 3. SK channel parameters and equations. Parameters controlling SK channel activity are based on a timescale of milliseconds, where applicable.

**Parameter**	**Value**
p1	1.01×10^{-1}
p2	2.73×10^{-1}

p3	2.96
p4	2.00×10^{-1}
p5	2.79×10^{-1}
p6	-8.69×10^{1}
p7	6.36×10^{-3}
p8	5.23
p9	3.21×10^{-4}
g_{SK}	3.04×10^{-2}
F_j	1.10×10^{-1}
E_K	2.67×10^{1}
M_{ref}	1.05×10^{2}
**SK channel equations**	
$\varepsilon_{SKj} = \frac{1}{1 + \exp\left(\frac{log_{10}(p9) - log_{10}([Ca^{2+}]_j)}{0.3}\right)}$	
$\varepsilon_{SKs} = \frac{1}{1 + \exp\left(\frac{log_{10}(p9) - log_{10}([Ca^{2+}]_{sl})}{0.3}\right)}$	
$\eta_{SKv} = \frac{p2}{1 + \exp((V_m - E_K + p3) \cdot p4)} + \frac{p5}{1 + \exp((-(V_m - E_K + p6)) \cdot p7)}$	

Comment: Making a concrete version of the code available in a persistent and citable way, I could not clearly identify the relevant code from the given link. GitHub/Zenodo integration is a convenient way of doing this.

Response: We have made the code and scripts publicly available via the Heijman Lab GitHub repository. This allows full version history tracking, which supports reproducibility and long-term accessibility of our model code and scripts. Furthermore, an archived, citable snapshot of the code is accessible via Zenodo.

We have added this relevant point to the methods of the revised manuscript (blue) at lines 289-291: The model code, optimization scripts, and data are available online at https://github.com/HeijmanLab, and an archived, citable snapshot of the specific version used in this study is accessible via Zenodo at https://doi.org/10.5281/zenodo.15494834.

Reviewers' comments:

Reviewer #1

Comment: Previous publications have shown that intracellular Ca plays dual roles in the SK channel regulation. Rapid pacing promotes SK channel membrane-localization, therefore this model studied a physiologically relevant question. However, both the cardiomyocyte and the SK trafficking component are based on previously reported models, the reviewer is therefore concerned of the limited novelty of this work.

Response: We thank the reviewer for raising this point. While our model builds on previous work, notably our recent model of K_V11.1 trafficking in human ventricular cardiomyocytes, it introduces key innovations specific to SK channels. We developed a novel Ca^{2+}-dependent sensor to capture the rate-dependent regulation of SK-channel trafficking and, for the first time, incorporated the downstream electrophysiological effects into 2D tissue simulations to assess their role in arrhythmogenesis over extended timescales. To our knowledge, no prior model has combined these features for SK channels or ion-channel trafficking in general. In addition to these methodological advances, our work provides novel insights into the dual effects of SK-channel inhibition, indicating that it reduces reentry duration but may promote ectopy, suggesting a potential effect on AF burden rather than a complete freedom of AF episodes. In the revised version we have further expanded these novel insights into the antiarrhythmic effects of SK-channel inhibition by comparing it to other pharmacological interventions, based on suggestions from the reviewers.

Comment: SK channel activation depends on the increase in intracellular Ca2+ of the cardiomyocytes on a beat-to-beat basis. However, the Ca2+ handling is oversimplified in this model with a fixed subsarcolemmal Ca2+ concentration. The Ca2+-sensor of SK channels, which regulates the SK trafficking and activation, likely senses local Ca2+ and may be altered at higher frequency. Therefore, the model may not be able to predict SK channel function accurately. The author should discuss how the fixed cytosolic Ca2+ concentration affect the Ca2+-sensor function and consider incorporating more detailed Ca2+ buffering kinetics into the model.

Response: We thank the reviewer for this comment and fully agree that local Ca^{2+} handling is important for the regulation of SK channels. We used buffered Ca^{2+} during model calibration (Figure 1C, right panel; and Figure 2, all black data points) to match experimental conditions, which were also conducted under buffered Ca^{2+} (500 nmol/L free Ca^{2+} using appropriate intrapipette EGTA and total Ca^{2+} concentrations). A key strength of our approach is that, following calibration, the model can be used to simulate responses to free Ca^{2+}, allowing exploration of more physiologically relevant scenarios, including beat-to-beat and frequency-dependent effects. These simulations with free Ca^{2+} were included in Figure 1C (left panel), Figure 2 (red data points), and throughout all subsequent figures in the original manuscript. Importantly, in these simulations SK channels sense local Ca^{2+} levels in different compartments (see our response to the next comment below).

Comment: SK channels have previously been shown to be coupled to L-type Ca channel (LTCC) and ryanodine receptors (RyR). The authors should consider integrating LTCC and RyR coupling into the model.

Response: We thank the reviewer for this suggestion. The gating of the SK-channel in our model is based on our recent publication (Heijman *et al.*, 2023). This model considers two populations of SK channels, those located in the subsarcolemmal space and those located in the junctional compartment. The latter group would represent those that are close to LTCC and RyR channels. The distinction between both groups is based on previous publications highlighting a less prominent interaction between LTCC and RyR in atrial cardiomyocytes compared to ventricular cardiomyocytes due to a relative paucity of T-tubules in the former. In our novel trafficking model, the Ca^{2+} sensor that controls forward trafficking is driven by subsarcolemmal Ca^{2+}, which indirectly reflects contributions from both LTCC and RyR activity. While we acknowledge the importance of spatial coupling in cardiomyocyte Ca^{2+} handling, at present the exact molecular mechanisms controlling the experimentally observed Ca^{2+} dependence of SK-channel trafficking and their subcellular localization remain unknown. Based on the localization of SK channels in the original model and the less extensive T-tubular network in atrial cardiomyocytes, we chose to use subsarcolemmal Ca^{2+} levels for the trafficking model. Importantly, this localization retains a strong connection with LTCCs, with a 70% block of LTCCs producing a pronounced reduction in SK-channel trafficking and gating (Figure 2B-C), consistent with our previous experimental results with the LTCC blocker nifedipine (Heijman *et al.*, 2023). Nevertheless, we acknowledge that our phenomenological approach might need to be refined when more experimental data about the molecular mechanisms of Ca^{2+}-dependent regulation of SK-channel trafficking and/or subcellular localization in human atrial cardiomyocytes become available.

We have added this relevant point to the discussion of the revised manuscript (blue) at lines 512 - 518: Nonetheless, spatial coupling was implicitly considered, as the Ca^{2+} sensor and SK-channel forward trafficking were controlled by subsarcolemmal Ca^{2+}, which is modulated by LTCC and RyR2 activity. This connection to LTCCs is supported by the Nifedipine simulations, which show a similar reduction in SK-channel trafficking and gating as observed experimentally (Heijman *et al.*, 2023). While phenomenological, this approach can be refined as more experimental data on Ca^{2+}-dependent regulation and SK-channel localization in human atrial cardiomyocytes become available.

Comment: Calcium affinity and Hill coefficients are also key parameters of the channel trafficking, the author should perform sensitivity analysis on these parameters.

Response: We thank the reviewer for this suggestion and have added a sensitivity analysis of these parameters (Reviewer Figures 1-2). In brief, a smaller K_m increases Ca_{dep}, as the sensor becomes more responsive to Ca^{2+}, requiring less Ca^{2+} to activate. This effect is further amplified at high pacing rates (e.g., 5 Hz), where increased Ca^{2+} loading leads to higher subsarcolemmal Ca^{2+} concentrations and thus stronger activation. At lower pacing frequencies (e.g., 1 Hz), the Hill coefficient significantly

affects SK channel activation by modulating the steepness of the Ca^{2+}-dependent response. However, at high frequencies (e.g., 5 Hz), the Ca^{2+} sensor approaches saturation, reducing the influence of the Hill coefficient on downstream variables such as membrane expression, sub-membrane accumulation, and f-trap. We have chosen not to include these analyses in the revised manuscript because Reviewer 3 suggested a population of models (POM)-based sensitivity analysis that also includes these parameters; the results of that analysis are presented in the revised manuscript.

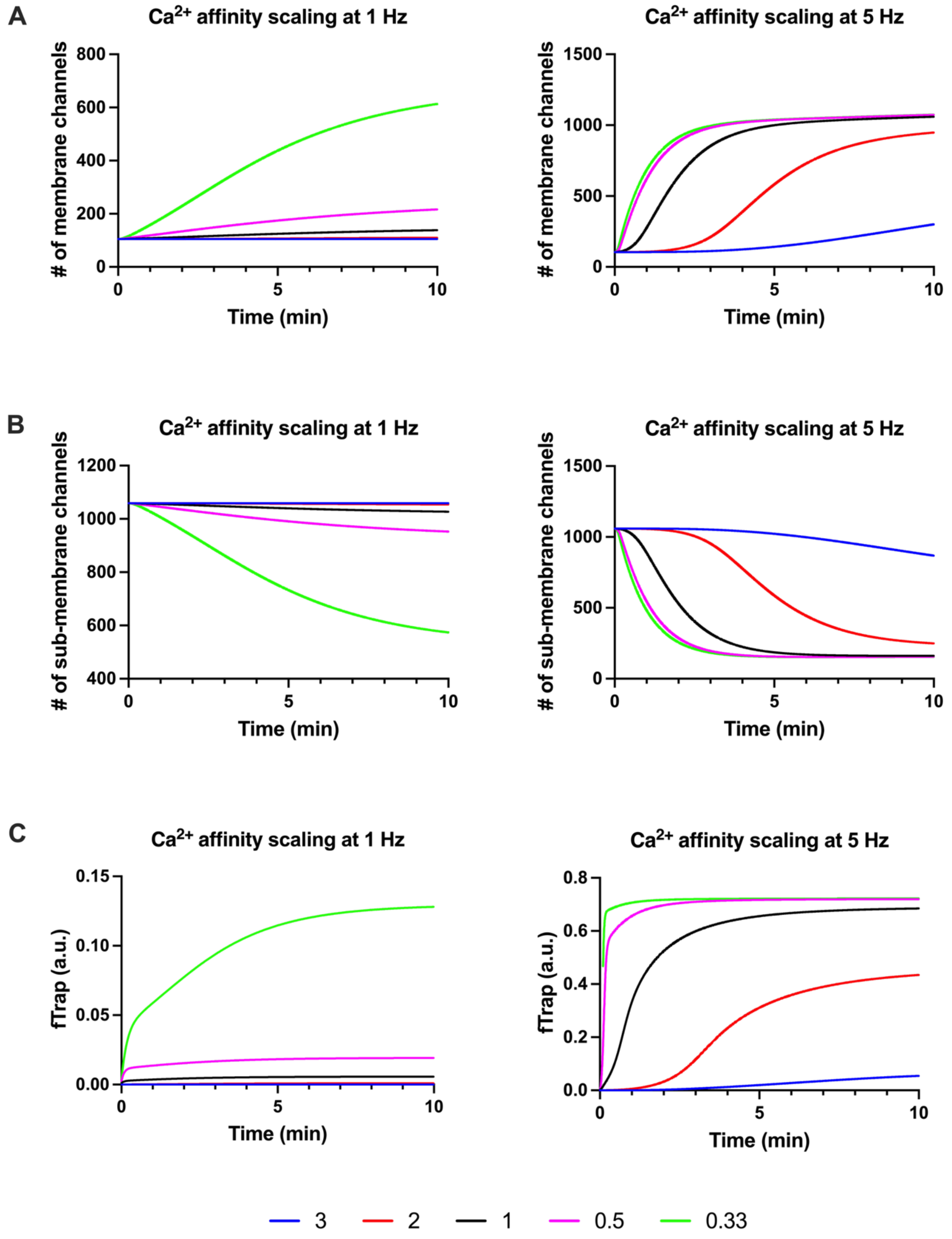

Reviewer Figure 1. Sensitivity of SK channel behavior to changes in the Ca^{2+}affinity of the Ca^{2+} sensor (K_m). *A,* Effect of scaling K_m (3-, 2-, 1-, 0.5-, or 0.33-fold) on the number of SK channels in the membrane and their dynamics after 10 minutes of triangular voltage-clamp stimulation at either 1 Hz (left panel) or 5 Hz (right panel). *B,* Corresponding changes in the number of sub-membrane SK channels under identical conditions. *C,* Activation and dynamic behavior of the Ca^{2+} sensor at different K_m values, highlighting the rate-dependent differences between 1-Hz (left panel) and 5-Hz (right panel) stimulations.

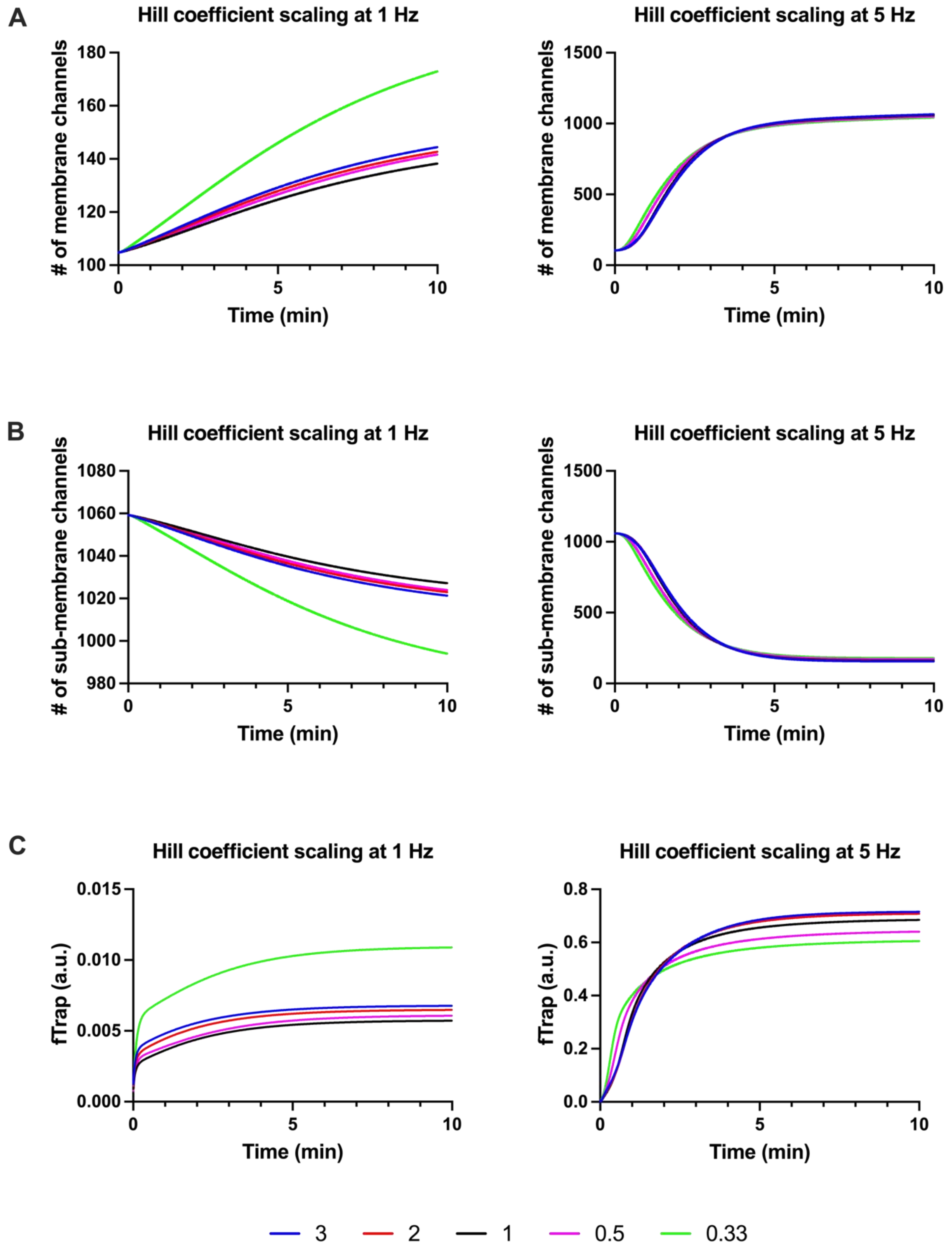

Reviewer Figure 2. Sensitivity of SK channel behavior to changes in the Hill coefficient of the Ca^{2+} sensor. *A,* Effect of scaling the Hill coefficient (3-, 2-, 1-, 0.5-, or 0.33-fold) on the number of SK channels in the membrane and their dynamics after 10 minutes of triangular voltage-clamp stimulation at either 1 Hz (left panel) or 5 Hz (right panel). *B,* Corresponding changes in the number of sub-membrane SK channels under identical conditions. *C,* Activation and dynamic behavior of the Ca^{2+} sensor at different Hill coefificient values, highlighting the rate-dependent differences between 1-Hz (left panel) and 5-Hz (right panel) stimulations.

Comment: The 2D model provides valuable insights into the role SK channels in arrhythmogenesis, however, the model assumed a homogenous tissue structure, which could affect the accuracy of reentry.

Response: We agree with the reviewer that real cardiac tissue is highly heterogeneous, which can significantly influence reentry dynamics. We have extended and adapted our *Discussion* at lines 626 - 630 accordingly (blue):

"Additionally, the 2D tissue model used in this study was homogeneous, whereas real cardiac tissue exhibits significant physiological heterogeneity, which could influence patterns of electrical activation. In particular, patchy distributions of conductivity or excitability, arising from fibrosis, ion channel remodeling, or cell-cell uncoupling, can anchor reentrant circuits or promote wavebreak. These phenomena are not captured in our homogeneous model. Such factors may affect the generalizability of our findings to more complex, realistic scenarios."

Reviewer #3

Comment: The model presents significant strengths, particularly its multi-scale development and detailed exploration of ISK channel dynamics in the context of AF. To build further upon this solid foundation, the authors might consider extending their modeling approach to explore how variations in other clinically relevant ionic currents, targeted by existing AF therapies, can influence the outcomes observed with ISK modulation/2D simulation. For instance, evaluating the interplay between ISK inhibition and commonly used anti-arrhythmic drugs could provide insights into synergistic or additive therapeutic effects, potentially uncovering novel benefits or limitations of current treatments.

Response: We thank the reviewer for this insightful comment. To explore potential interactions between I_{SK} inhibition and other potentially relevant ionic current blockers, we performed additional cellular-level simulations comparing I_{SK} block with I_{Kur}, I_{Kr}, I_{K2P} block and combined I_{SK} + I_{K2P} block under different pacing rates. I_{Kur} block was implemented using the formulation by Grandi et al. (2011) for the I_{Kur} inhibitor AVE0118 at 10 µmol/L. This approach captures both the concentration- and frequency-dependent characteristics of I_{Kur} inhibition. I_{Kr} block was modeled using 3.8 nmol/L dofetilide to simulate the effects of a clinically relevant dose (Meier et al., 2023). SK-channel block was simulated as an 80% reduction in I_{SK}, similar to the 2D tissue level simulations performed in the original version of our manuscript. Finally, the I_{K2P} block was inspired on data from Heijman et al. (2023) and Dasí et al. (2024), which both simulated a complete block of the K2P channel. In our simulations, we chose to match the SK-channel block percentage for K2P (i.e., simulating an 80% reduction in I_{K2P}) to facilitate a comparison to the synergistic effects of K2P and SK channel block observed by Dasí et al.

The APD_{90} was 223 ms under control conditions after 1 Hz pacing for 10 minutes. In the presence of I_{Kur} block, I_{Kr} block, I_{SK} block, or I_{K2P} block, APD was 234, 225, 263, and 239 ms, respectively (new Figure 9, also shown below). The combined effects of I_{K2P} and I_{SK} block resulted in an APD_{90} of 291 ms. After an additional 10 minutes of 5 Hz pacing, APD_{90} was 61 ms in control, 64 ms with I_{Kur} block, 60 ms with I_{Kr} block, 146 ms with I_{SK} block, 62 ms with I_{K2P} block, and 160 ms with combined block of I_{SK} and I_{K2P} (Reviewer Figure 2B). These results indicate that SK-channel inhibition has the most pronounced APD_{90}-prolonging effect at both slow and fast pacing rates, suggesting a more pronounced antiarrhythmic effect than I_{Kur} or I_{Kr} block. These data support our focus on the antiarrhythmic effects of SK-channel inhibition in 2D simulations in the last figure of our manuscript.

We added the following section to the results at lines 425 - 445 (blue):

Comparison between SK-channel block and inhibition of other K^+ channels

To explore potential interactions between I_{SK} block and inhibition of other potentially relevant K^+ currents, we performed additional cellular simulations comparing I_{SK} block with I_{Kur}, I_{Kr}, I_{K2P} and combined I_{K2P} + I_{SK} inhibition during 1 Hz and 5 Hz pacing. I_{SK} block was implemented as an 80% reduction in current amplitude, while I_{Kur} block was implemented using the AVE0118 formulation from Grandi et al. (2011) at 10 µmol/L to capture both its concentration and frequency dependence (Grandi et al., 2011). A

clinically relevant dose of dofetilide (3.8 nmol/L) was used to simulate the effects of I_{Kr} block, resulting in a 30% reduction in I_{Kr} (Meier et al., 2023). Finally, I_{K2P} block was modeled as an 80% reduction to enable direct comparison with the degree of SK-channel block and the previously reported synergistic effects of combined I_{SK} and I_{K2P} block (Heijman et al., 2023; Dasí et al., 2024).

The APD_{90} was 223 ms under control conditions after 1 Hz pacing for 10 minutes. In the presence of I_{Kur}, I_{Kr}, I_{SK}, or I_{K2P} block, APD_{90} was 234, 225, 263, and 239 ms, respectively (Figure 9A). The combined effects of I_{K2P} and I_{SK} block resulted in an APD_{90} of 291 ms. After an additional 10 minutes of 5-Hz pacing, APD_{90} was abbreviated to 61 ms in control, 64 ms with I_{Kur} block, 60 ms with I_{Kr} block, 146 ms with I_{SK} block, 62 ms with I_{K2P} block, and 160 ms with combined block of I_{SK} and I_{K2P} (Figure 9B). These results indicate that SK-channel inhibition has the most pronounced APD_{90}-prolonging effect at both slow and fast pacing rates, suggesting a potentially stronger antiarrhythmic effect than I_{Kur} or I_{Kr} block. Furthermore, the combined effects of I_{SK} and I_{K2P} block resulted in a synergistic APD_{90} prolongation under both pacing conditions, consistent with previous observations (Dasí et al., 2024).

We added the following section to the discussion at lines 589 - 598 (blue):
A recent *in silico* study by Dasí et al. (2024) demonstrated that I_{SK} and I_{K2P} block alone cardioverted 33% and 43% of virtual AF patients, respectively, while their combined application increased cardioversion rates to 82%. Our cellular simulations similarly showed a greater-than-additive APD_{90} prolongation with combined I_{SK} and I_{K2P} block, particularly under AF-mimicking conditions. Together, these findings suggest that a multi-target pharmacological approach involving SK-channel inhibition may represent a promising strategy for rhythm control in AF. Such an approach may also limit the ectopic activity-promoting effects of SK-channel inhibition that we have identified in our simulations (e.g., via concomitant inhibition of Na^+ channels).

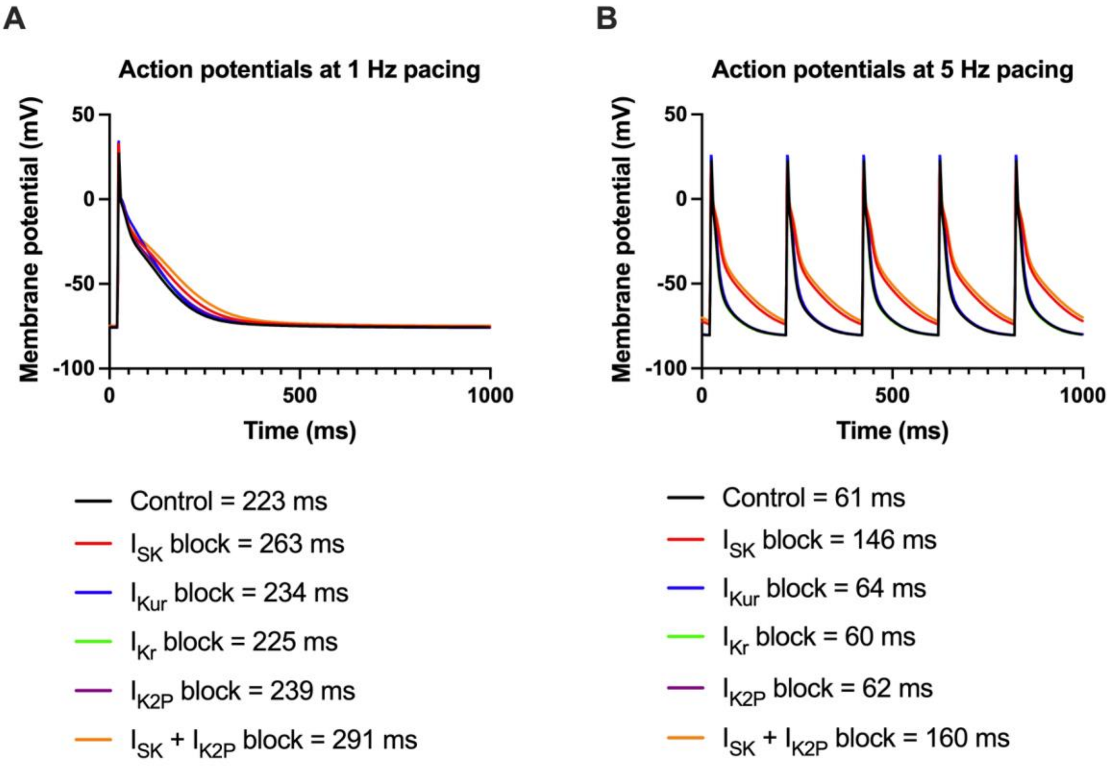

Figure 9. Action potentials under different drug block conditions and pacing frequencies. A, Action potentials and corresponding action potential duration (APD_{90}, shown in the legend) of the last beat during 10 minutes of pacing at 1 Hz under control conditions, I_{SK} block, I_{Kur} block, I_{Kr} block, I_{K2P} block, or I_{SK} + I_{K2P} block. B, Similar to panel A for the last second of 5 Hz pacing for 10 minutes, after 10 minutes of initial pacing at 1 Hz, for all drug conditions.

Comment: A population-based modeling framework could substantially enrich the robustness and translational potential of key findings. By exploring variability across simulated patient populations, the authors could identify factors that enhance or diminish vulnerability to AF related to ISK trafficking dynamics. Such an approach would also help pinpoint conditions (such as specific disease states or genetic variants) that may uniquely influence ISK channel trafficking and thus modify arrhythmic risk.

Response: We thank the reviewer for this valuable suggestion. To assess the robustness and variability of I_{SK}-related dynamics, we implemented a population-of-models (PoM) based modeling framework in which SK-channel trafficking and Ca^{2+} sensor parameters were randomly varied using a normal distribution (σ = 0.1), following the approach of Heijman et al. (2023). Given the computational cost of long simulations (10 minutes at 1 Hz followed by 10 minutes at 5 Hz), we only generated 100 distinct parameter sets and calculated the impact of each parameter on APD at fast and slow rates. We repeated the simulation protocol five times (e.g., 5 times 100 distinct parameter combinations) to compute the mean and standard deviation of the impact of each parameter for each condition.

Reviewer Figure 3 (New Figure 6) demonstrates that, after 10 minutes at 1 Hz, membrane SK-channel levels are primarily influenced by fast regulatory processes, such as forward and backward trafficking rates and Ca^{2+}-sensor kinetics. After an additional 10 minutes at 5 Hz, the variability in functional SK-channel expression becomes dominated by slower processes, including protein production and degradation. This highlights how dynamic regulation over different timescales may shape individual susceptibility to AF under variable physiological or pathological conditions. During 1 Hz pacing, the relative impact of each parameter on APD parallels that of the SK-channel levels (albeit in opposite direction since an increase in SK-channel levels produces a reduction in APD). By contrast, during 5 Hz pacing, the absolute impact of all parameters on APD is smaller, since the average APD is much shorter under these conditions. Moreover, the parameters controlling the Ca^{2+} sensor have the most pronounced influence, highlighting the importance of Ca^{2+}-dependent SK-channel trafficking in shaping APD during 5 Hz pacing.

The reviewer raises an interesting point about using the variability across simulated patient populations to identify factors that enhance or diminish vulnerability to AF related to I_{SK} trafficking dynamics. Classical PoM approaches employ variability in maximal conductances (implicitly reflecting cell-to-cell differences in expression levels) to assess a channel's contribution to an outcome of interest. However, in our framework, I_{SK} is not determined by a predefined maximal conductance, but instead emerges from the time-dependent regulation of membrane channel density, making the concept of cell-to-cell variability in maximal conductance challenging. Moreover, traditional PoM approaches may fail to capture key dynamic processes such as the dynamic redistribution of SK channels to cellular compartments during tachypacing, which can substantially influence APD and arrhythmia vulnerability. For example, an increase in forward trafficking may be associated with an increased AF vulnerability at slow rates, but may not change the vulnerability at rapid rates (if the total number of channels does not change and all channels trafficked to the membrane). Thus, although PoM remains useful for identifying parameter influence on outcome measures such as APD_{90} (used here as a proxy for AF risk), it is less suitable for capturing the mechanistic underpinnings of SK trafficking-related variability and its impact on arrhythmogenesis.

We added the following section to the results at lines 373 - 386 (blue):
In addition, we implemented a population-based modeling framework in which SK-channel trafficking and Ca^{2+}-sensor parameters were randomly varied, following the approach of Heijman et al. (2023). We generated 100 distinct parameter sets and repeated the simulation protocol five times to assess the impact of each parameter on membrane channels (Figure 6A) and APD (Figure 6B) at both slow and fast pacing rates. At 1 Hz, membrane channel levels were primarily driven by fast trafficking kinetics and Ca^{2+}-sensor dynamics, whereas at 5 Hz, slower processes such as protein production and degradation became the dominant contributors. During 1 Hz pacing, the relative impact of each parameter on APD parallels that of the SK-channel levels (albeit in opposite direction since an increase in SK-channel levels produces a reduction in APD). By contrast, during 5 Hz pacing, the absolute impact of all parameters on APD is smaller, since the average APD is much shorter and the parameters controlling the Ca^{2+} sensor have the most pronounced influence,

supporting an important role of Ca^{2+}-dependent SK-channel trafficking in shaping APD during 5 Hz pacing.

We added the following section to the discussion at lines 633 - 638:
Furthermore, we employed a population of models approach to assess the influence of individual model parameters on APD. However, predicting the impact of these findings on arrhythmogenesis is challenging. For example, a redistribution of channels from the submembrane to the plasma membrane state may increase reentry vulnerability at baseline by causing APD abbreviation, but would reduce the proarrhythmic consequences of tachycardia-induced trafficking promotion.

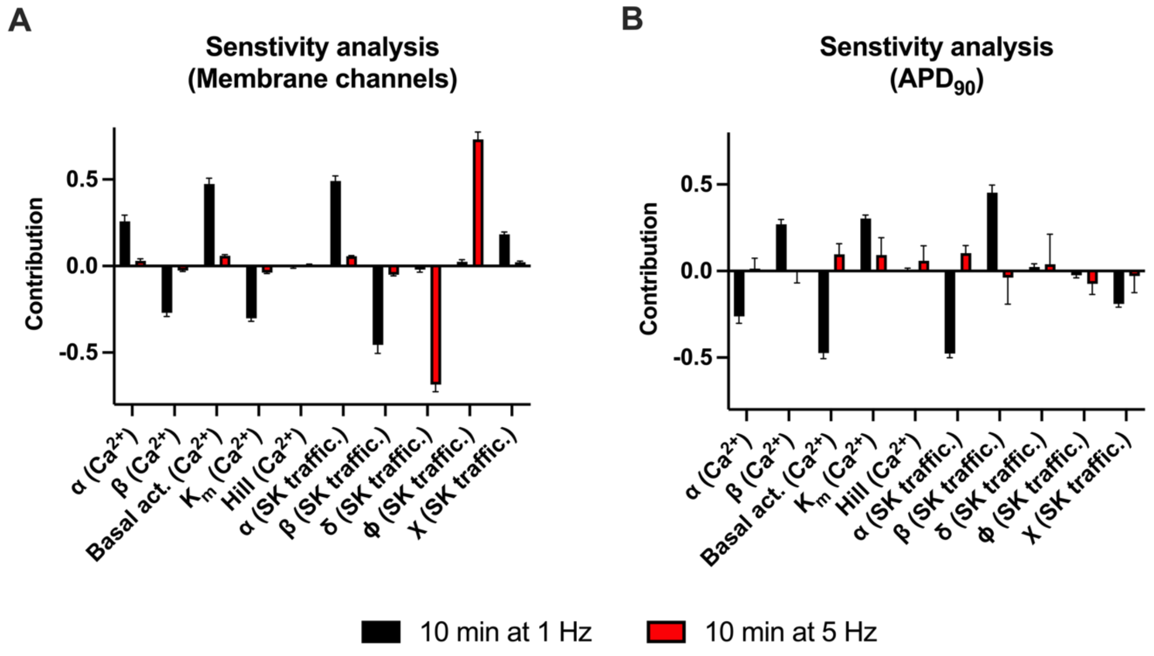

Figure 6. Population of Models (PoM) analysis of SK trafficking and Ca^{2+} sensor parameters. SK-channel trafficking and Ca^{2+} sensor parameters were randomly varied using a normal distribution ($\sigma = 0.1$) to generate 100 distinct parameter sets. This protocol was repeated five times to evaluate the impact of each parameter on SK membrane channels (panel A) and APD (panel B) after 10 minutes at 1 Hz (black) followed by 10 minutes at 5 Hz pacing (shown in red), and to compute the mean and standard deviation of their influence under each condition.

Comment: Overall, the current manuscript provides important foundational knowledge and conceptual innovation. Considering some of these suggestions or applying the model in another way to generate new knowledge would improve the manuscript's impact by providing insights into AF mechanisms and therapy. These simulations could yield new insights into different therapeutic strategies/risks, thereby significantly enhancing the clinical implications of this important model.

Response: We appreciate the reviewer's thoughtful feedback and recognition of the conceptual novelty of this work. We agree that extending the model to explore therapeutic strategies and disease-specific alterations presents an exciting opportunity for future studies to further enhance the translational relevance of this

framework. Based on the reviewer's suggestion, we have therefore added the drug-effect simulations from the comment above to the main manuscript.

Dear Professor Heijman,

Re: JP-RP-2025-288659R1 "**Computational Modeling of Pro- and Antiarrhythmic Effects of Atrial High Rate-Dependent Trafficking of Small-Conductance Ca^{2+}-Activated K^{+} Channels**" by Stefan Meier, Dobromir Dobrev, Paul GA Volders, and Jordi Heijman

We are pleased to tell you that your paper has been accepted for publication in The Journal of Physiology.

The last Word (or similar) version of the manuscript provided will be used by the Production Editor to prepare your proof. When this is ready you will receive an email containing a link to Wiley's Online Proofing System. The proof should be thoroughly checked and corrected as promptly as possible.

Authors should note that it is too late at this point to offer corrections prior to proofreading. Major corrections at proof stage, such as changes to figures, will be referred to the Editors for approval before they can be incorporated. Only minor changes, such as to style and consistency, should be made at proof stage. Changes that need to be made after proof stage will usually require a formal correction notice.

All queries at proof stage should be sent to: TJP@wiley.com.

Following copyediting and typesetting the accepted version will be published online.

Yours sincerely,

Bjorn Knollmann
Senior Editor
The Journal of Physiology

IMPORTANT POINTS TO NOTE FOLLOWING YOUR PAPER'S ACCEPTANCE:

If you would like to receive our 'Research Roundup', a monthly newsletter highlighting the cutting-edge research published in The Physiological Society's family of journals (The Journal of Physiology, Experimental Physiology, Physiological Reports, The Journal of Nutritional Physiology and The Journal of Precision Medicine: Health and Disease), please click this link, fill in your name and email address and select 'Research Roundup':
https://www.physoc.org/journals-and-media/membernews

• TRANSPARENT PEER REVIEW POLICY: To improve the transparency of its peer review process, The Journal of Physiology publishes online as supporting information the peer review history of all articles accepted for publication. Readers will have access to decision letters, including Editors' comments and referee reports, for each version of the manuscript as well as any author responses to peer review comments. Referees can decide whether or not they wish to be named on the peer review history document.

• You can help your research get the attention it deserves! Check out Wiley's free Promotion Guide for best-practice recommendations for promoting your work at: www.wileyauthors.com/eeo/guide. You can learn more about Wiley Editing Services which offers professional video, design, and writing services to create shareable video abstracts, infographics, conference posters, lay summaries, and research news stories for your research at: www.wileyauthors.com/eeo/promotion.

• IMPORTANT NOTICE ABOUT OPEN ACCESS: To assist authors whose funding agencies mandate public access to published research findings sooner than 12 months after publication, The Journal of Physiology allows authors to pay an Open Access (OA) fee to have their papers made freely available immediately on publication.

The Corresponding Author will receive an email from Wiley with details on how to register or log-in to Wiley Authors Services where you will be able to place an order.

• You can check if your funder or institution has a Wiley Open Access Account here: https://authorservices.wiley.com/author-resources/Journal-Authors/licensing-and-open-access/open-access/author-compliance-tool.html.

EDITOR COMMENTS

Reviewing Editor:

Your manuscript was assessed again and all expert referees confirmed that their concerns have been addressed sufficiently.

Senior Editor:

The manuscript is now acceptable for publication. Thank you for your excellent contribution!

REFEREE COMMENTS

Referee #1:

The authors have been responsive to prior comments and suggestions. The reviewer appreciates the fact that the limitations section has been added that will be extremely helpful to the readers and future studies. There are no additional concerns.

Referee #3:

The authors have responded to each of my concerns. This manuscript is expected to have a significant impact on the field.
